# Mitochondrial RNA stimulates beige adipocyte development in young mice

**DOI:** 10.1038/s42255-022-00683-w

**Published:** 2022-11-28

**Authors:** Anh Cuong Hoang, László Sasi-Szabó, Tibor Pál, Tamás Szabó, Victoria Diedrich, Annika Herwig, Kathrin Landgraf, Antje Körner, Tamás Röszer

**Affiliations:** 1 Institute of Neurobiology, Ulm University, Ulm, Germany; 2 Institute of Pediatrics, Clinical Centre, Faculty of Medicine, University of Debrecen, Debrecen, Hungary; 3 Center for Pediatric Research, University Hospital for Children and Adolescents, University of Leipzig, Leipzig, Germany; 4 Helmholtz Institute for Metabolic, Obesity and Vascular Research (HI-MAG) of the Helmholtz Center München at the University of Leipzig and University Hospital Leipzig, Leipzig, Germany

**Keywords:** Fat metabolism, Antimicrobial responses, Mitochondria

## Abstract

Childhood obesity is a serious public health crisis and a critical factor that determines future obesity prevalence. Signals affecting adipocyte development in early postnatal life have a strong potential to trigger childhood obesity; however, these signals are still poorly understood. We show here that mitochondrial (mt)RNA efflux stimulates transcription of nuclear-encoded genes for mitobiogenesis and thermogenesis in adipocytes of young mice and human infants. While cytosolic mtRNA is a potential trigger of the interferon (IFN) response, young adipocytes lack such a response to cytosolic mtRNA due to the suppression of IFN regulatory factor (IRF)7 expression by vitamin D receptor signalling. Adult and obese adipocytes, however, strongly express IRF7 and mount an IFN response to cytosolic mtRNA. In turn, suppressing IRF7 expression in adult adipocytes restores mtRNA-induced mitobiogenesis and thermogenesis and eventually mitigates obesity. Retrograde mitochondrion-to-nucleus signalling by mtRNA is thus a mechanism to evoke thermogenic potential during early adipocyte development and to protect against obesity.

## Abstract (web summary)

Hoang et al. show that mitochondrial RNA in young beige adipocytes triggers mitobiogenesis and thermogenesis, while an interferon response is avoided through vitamin D-mediated suppression of IRF7 expression.

## Main

Childhood obesity is a serious public health crisis today and is associated with an increased risk of adult obesity and diabetes, which is projected to affect ~58% of the world’s adult population by 2030 (refs. ^1–4^). Storing fat in adipocytes is necessary for metabolic and endocrine health; however, excess fat accumulation accounts for the development of metabolic diseases^1,2,4–6^. Infancy and early childhood (before 5.5 years of age) are critical periods that determine adipocyte number and fat accumulation, thereby also determining future obesity^7–9^. For example, an accelerated rate of body weight gain and overweight during the first year of life and in early childhood increase the probability of obesity and obesity-associated diseases in adulthood^1,2,5,6^. Signals that control adipocyte development in early postnatal life thereby have a strong potential to trigger obesity; however, these signals are still poorly understood.

The human foetus accumulates fat in subcutaneous adipose tissue depots at the last trimester, and fat becomes a relevant energy source at birth and in early postnatal life^10–12^. The foetus has a carbohydrate-based metabolism, which shifts to lipid-rich nutrition in the form of breastfeeding or formula feeding at birth^10–12^. The subcutaneous adipose tissue of a newborn is actively engaged in lipolysis and beta oxidation of fatty acids to generate energy^10–12^. Mammals are homoeothermic animals; hence the foetus develops in a thermally stable environment in the womb, without the demand of investing energy into the maintenance of its own core body temperature. However, this scenario rapidly changes at birth, when the newborn is challenged with a hypothermic environment without the ability to perform shivering thermogenesis. The large energy demand of homoeothermy is covered by burning off fat as heat in the adipose tissue, in a process often called non-shivering thermogenesis or adaptive thermogenesis^13^. It is estimated that the heat produced in a human term infant is generated mostly by fat (64%) catabolism in non-shivering thermogenesis^14,15^. The responsible thermogenic adipocytes produce heat from uncoupled oxidative phosphorylation in their extensive mitochondrial network, using lipids as metabolic fuel^12,16^. Thermogenic adipocytes are distributed in the subcutaneous fat depots of a newborn^17^ and are still detectable in early childhood^12,16^. By contrast, adult humans have shivering thermogenesis, and subcutaneous adipose tissue depots function as thermal insulators and long-term storage sites of fat^17^. Adipocytes of subcutaneous fat depots in adults are therefore poor in mitochondria and accumulate lipids instead of metabolizing them to energy or heat^18^.

Thermogenic adipocytes in human subcutaneous adipose tissue progressively disappear by the age of 5–7 years, when adipose tissue expansion occurs as a physiological process^1,2,7^. Subcutaneous fat depots of young mice are rich in thermogenic adipocytes, which disappear by weaning age^16^. This trait of young mice resembles the development of thermogenic fat cells in humans, making them suitable models to study mechanisms of early-life human adipocyte development. Recent studies suggest that premature loss of thermogenic fat cells is associated with early onset of adipose tissue expansion, and these two factors together may lead to childhood obesity^1,2,7,12,16^. It is plausible that delaying or reverting the metabolic shift from fat catabolism and thermogenesis to fat storage has therapeutic potential in obesity prevention^1,2,12,16^.

The conversion of fat into energy and heat requires an extensive mitochondrial network^19,20^, and, accordingly, adipocytes of the subcutaneous adipose tissue are rich in mitochondria in early postnatal life^10,15^. However, the abundance of mitochondria is associated with a potentially inflammation-provoking efflux of mtDNA to the cytosol^21^. Due to its endosymbiotic origin, mtDNA resembles prokaryote-type DNA and eventually triggers an IFN response^22–25^. Obese adipocytes produce IFNs, and IFNs eventually trigger metabolic diseases^26,27^. IFNs may damage the mitochondrial network and the capacity for fat oxidation and thermogenesis and trigger metabolic inflammation and insulin resistance^28,29^. It has been shown that activation of the stimulator of IFN-response genes (STING) pathway (a major cytosolic DNA-sensing pathway) worsens obesity and abrogates the thermogenic programme in adipocytes^30^. In turn, inhibition of mtDNA efflux into the adipocyte cytosol effectively reduces obesity-associated inflammation and insulin resistance^31^.

The efflux of mtDNA is inevitably associated with the release of mtRNA, which contains double-stranded RNA (dsRNA) motifs, strong inducers of the IFN response^22–25^. Given the abundance of mitochondria in adipocytes of young mice and human infants^32^, we asked whether these cells have a unique signalling mechanism that supports their mitochondrial network by mitigating the IFN response to mtRNA. While exploring this, we unexpectedly found that cytosolic mtRNA activated mitochondrion-to-nucleus signalling in adipocytes, which stimulated expression of nuclear-encoded genes of mitobiogenesis and thermogenesis in young mice and human infants. The IFN response to mtRNA was lacking in young adipocytes, plausibly due to the suppression of IRF7 by vitamin D receptor (VDR) signalling. Adult and obese adipocytes, however, expressed IRF7 strongly, and they mounted an IFN response to cytosolic mtRNA, abrogating its signalling role to stimulate mitobiogenesis and thermogenesis. In turn, when we inhibited IRF7 expression with VDR activation and transfected adipocytes with mtRNA, we could effectively induce beige adipogenesis and mitigate obesity in mice. Retrograde mitochondrion-to-nucleus signalling by mtRNA is hence a new mechanism that controls early adipocyte development and protects against obesity.

## Results

### Young adipocytes are immune tolerant for cytosolic mtRNA

**Fig. 1:**
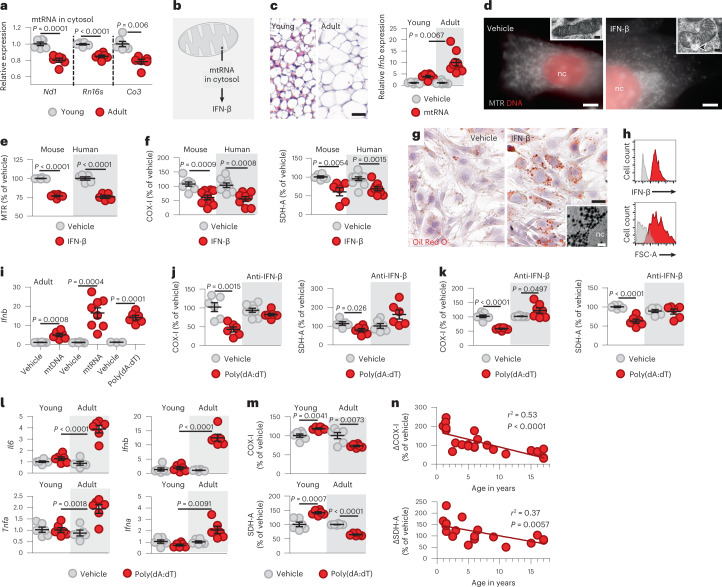
mtRNA does not trigger inflammation in young adipocytes. **a**, mtRNA in the cytosol of inguinal adipocytes of young (postnatal day 6) and adult (postnatal day 56) mice. *Rn16s* is also known as *Mt-Rnr2.* **b**, Cytosolic mtRNA potentially triggers an IFN response that is mediated by cytosolic RNA-sensor proteins, such as RIG-I and MDA5. **c**, Histology of young and adult mouse inguinal adipose tissue, hematoxylin and eosin (H&E); scale bar, 25 μm. Young and adult adipocytes of the same fat depot were transfected with mtRNA (2 μg ml^−1^, 18 h) and the transcript levels of *Ifnb* were measured. **d**, Effect of IFN-β on the mitochondrial network in adult mouse adipocytes. Cells were treated with vehicle or 125 IU ml^−1^ IFN-β for 18 h, and mitochondria were labelled with MitoTracker Red (MTR). Scale bar, 20 μm. Inserts show transmission electron microscopy images of mitochondria; scale bar, 20 nm. Arrow head indicates mitochondrial swelling. **e**, Effect of IFN-β on mitochondrial mass in adult mouse and human adipocytes, measured by MTR staining intensity. **f**, Effect of IFN-β on the level of mitochondrial encoded COX-I and nuclear-encoded SDH-A in adult mouse and human adipocytes. **g**, Effect of IFN-β on fat accumulation in mouse preadipocytes. Oil Red O labelling of lipid droplets. Scale bar, 50 μm. Insert shows electron microscopy of the lipid-rich cytoplasm. nc, nucleus. Scale bar, 20 nm. **h**, Flow cytometry histogram of human adipocyte IFN-β content and forward scatter area (FSC-A, proportional to cell size). **i**, Transcription of *Ifnb* in adult mouse adipocytes in response to 18-h-long transfection with 5 μg ml^−1^ mtDNA, mtRNA or poly(dA:dT). **j**, Levels of COX-I and SDH-A in adult mouse adipocytes following transfection with poly(dA:dT). Anti-IFN-β, presence of neutralizing antibody against IFN-β. **k**, Levels of COX-I and SDH-A in adult human adipocytes following transfection with poly(dA:dT). Anti-IFN-β, presence of neutralizing antibody against IFN-β. **l**, Transcription of IFN-response genes in young and adult mouse adipocytes in response to transfection with poly(dA:dT). *Ifna* is also known as *Ifna1*; *Tnfa* is also known as *Tnf*. **m**, Levels of COX-I and SDH-A in young and adult mouse adipocytes following transfection with poly(dA:dT). **n**, Correlation of donor age and the corresponding changes in COX-I and SDH-A levels in human adipocytes following transfection with poly(dA:dT). Data are represented as mean ± s.e.m. *n* = 6 (**a**), *n* = 6 young and *n* = 9 adult samples (**b**), *n* = 6 mouse and *n* = 5 human samples (**e**), *n* = 9 (**f**), *n* = 7 and 8 (for mtRNA) (**i**), *n* = 6 (**j**–**m**), *n* = 25 (for COX-I) and *n* = 24 (for SDH-A) biologically independent experiments (**n**). Assays shown in **d**,**g**,**h** were repeated four times. Statistical significance was determined using Student’s two-tailed unpaired *t-*test (**a**,**c**,**e**,**f**,**i**–**m**) or linear regression analysis (**n**). Source data

**Extended Data Fig. 1:**
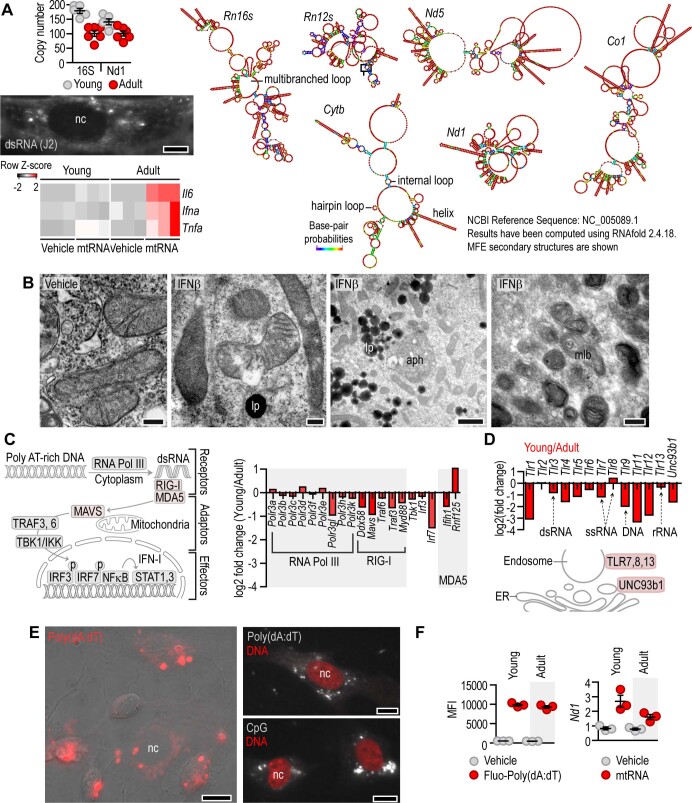
Recognition of cytosolic mtRNA in adipocytes. (**A**) *Top Left:* Copy number of mtDNA species in adipocytes of young and adult mice, N = 6 biologically independent samples. *Middle Left:* Immunostaining of dsRNA using J2 antibody in mouse adipocyte. nc: nucleus, Scale 10 μm. Assay was repeated three times. *Bottom Left:* Heat map of IFN-response genes in young and adult adipocytes transfected with vehicle or mtRNA (2 μg/ml, 18 h), N = 3 biologically independent samples. 80% confluent mouse adipocytes released 125 ± 20 IU/ml IFNβ in 4 h following mtRNA transfection. *Right:* Predicted minimum free energy (MFE) secondary structures of mtRNA species. Results were computed using ViennaRNA Package 2.0 and RNAfold 2.2.18, as described^88,89^. (**B**) Transmission electron microscopy of mitochondria, also shown in Fig. 1d. Assay repeated six times. Cells were treated with vehicle or IFNβ. lp: lipid droplet, aph: autophagosome, mlb: multilayered bodies. Scales from left to right: 1 μm, 20 nm, 1 μm, 1 μm. (**C**) Scheme of RIG-I/MDA5 signaling. RNA Pol III: RNA polymerase III, which can generate dsRNA from DNA templates, ultimately activating the RIG-I/MDA5 pathway. *Right:* Expression of RNA Pol III and RIG-I/MDA5 pathway genes in the adipose tissue of young mice. (**D**) *Mitochondrial RNA* stimulates human TLR8 (ref. ^90^) and triggers inflammation in mouse macrophages via TLR9 (ref. ^91^). Levels of *Tlr8* and *Tlr9* in young and adult subcutaneous adipose tissue in mouse. (**E**) Transfection of mouse adipocytes with rhodamine-conjugated poly (dA:dT) or CpG (a synthetic DNA sequence). Scale bar: 10 μm. Assay was repeated three times. (**F**) Mean fluorescence intensity of rhodamine-conjugated poly (dA:dT) in transfected cells, N = 3 biologically independent samples. Data are represented as mean ± SEM. Relative expression of mitochondrial *Nd1* in transfected cells. Source data

We found that inguinal adipocytes of young mice at postnatal day 6 contained more mitochondria and higher levels of cytosolic mtRNA than their adult counterparts (Fig. 1a and Extended Data Fig. 1a), suggesting that the cytosolic mtRNA level was proportional to the amount of mitochondria. Cytosolic mtRNA, due to the abundance of its dsRNA motifs (Extended Data Fig. 1a), triggers an IFN response^21,23–25,33,34^ (Fig. 1b). However, transfecting young (postnatal day 6) adipocytes with mtRNA failed to induce such a response (Fig. 1c and Extended Data Fig. 1a). In turn, adipocytes of adult mice (8 weeks of age) mounted a robust IFN response to cytosolic mtRNA (Fig. 1c and Extended Data Fig. 1a).

There are controversial findings about the effect of IFNs on mitochondrial energy and heat production^35–38^. To test the effect of IFN-β on adipocyte mitochondria, we cultured subcutaneous adipocytes of adult mice and non-obese, non-diabetic humans (age range, 16–17 years) and subjected them to IFN-β treatment. We used IFN-β at a concentration corresponding to the level of IFN-β secreted by adult mouse adipocytes in response to mtRNA transfection (Extended Data Fig. 1a). We found that IFN-β triggered mitochondrial damage and adipocytes exhibited signs of mitophagy (Fig. 1d and Extended Data Fig. 1b). IFN-β compromised mitochondrial mass (Fig. 1e) and reduced the level of the mitochondrial enzymes cyclooxygenase I (COX-I) and succinate dehydrogenase A (SDH-A) in both mouse and human adipocytes (Fig. 1f). Moreover, IFN-β treatment supported fat accumulation in preadipocytes (Fig. 1g). Coherently, hypertrophic human adipocytes had robust IFN-β expression (Fig. 1h). These effects of IFN-β on mitochondria^37^ and the robust expression of IFN-β by hypertrophic adipocytes agree with the findings that obesity development is associated with prominent expression of IFNs and IFN-stimulated genes (ISGs) in adipocytes^29,31^.

It is known that cytosolic mtRNA triggers *Ifnb* (*Ifnb1*) expression through cytosolic RNA sensors such as retinoic acid-inducible gene I (RIG-I) and RIG-I-like melanoma differentiation-associated protein 5 (MDA5)^24^. Accordingly, transfecting adult adipocytes with a synthetic ligand of cytosolic RNA sensors, so-called poly(deoxyadenylic-deoxythymidylic) acid (poly(dA:dT))^39^, increased *Ifnb* transcription to the same magnitude as cytosolic mtRNA (Fig. 1i). Cytosolic poly(dA:dT) is transcribed into RNA by the activity of RNA polymerase III, allowing stimulation of cytosolic RNA sensors^39^ (Extended Data Fig. 1c). Furthermore, cytosolic poly(dA:dT) mimicked the effect of IFN-β on adipocyte mitochondria in mice and humans (Fig. 1j,k). Conversely, an IFN-β-blocking antibody protected both mouse and human adipocytes from poly(dA:dT)-induced mitochondrial damage and slightly increased the levels of COX-I and SDH-A (Fig. 1j,k).

Importantly, neither cytosolic mtRNA (Fig. 1c) nor cytosolic poly(dA:dT) induced *Ifnb* transcription in young mouse adipocytes (Fig. 1l). Coherently, young mouse adipocytes were protected from mitochondrial damage induced by cytosolic poly(dA:dT) (Fig. 1m). This difference could not be explained by the absence of MDA5 or RIG-I in young adipocytes or by the inability of young adipocytes to be transfected with poly(dA:dT) (Extended Data Fig. 1c–f).

When we transfected adipocytes from human infants (aged 0–1 year), children (2–11 years) and adolescents (15–17 years) with poly(dA:dT), we found that adipocytes of infants and children were resistant to mitochondrial damage (Fig. 1n), similar to the adipocytes of young mice. Indeed, adipocytes of infants, similar to adipocytes of young mice, increased their COX-I and SDH-A levels following poly(dA:dT) transfection (Fig. 1m,n). In adipocytes of adolescents, however, cytosolic poly(dA:dT) reduced COX-I and SDH-A levels (Fig. 1n), mirroring poly(dA:dT) effects on adult mouse adipocytes (Fig. 1m).

### Young adipocytes have suppressed IRF7 expression

**Fig. 2:**
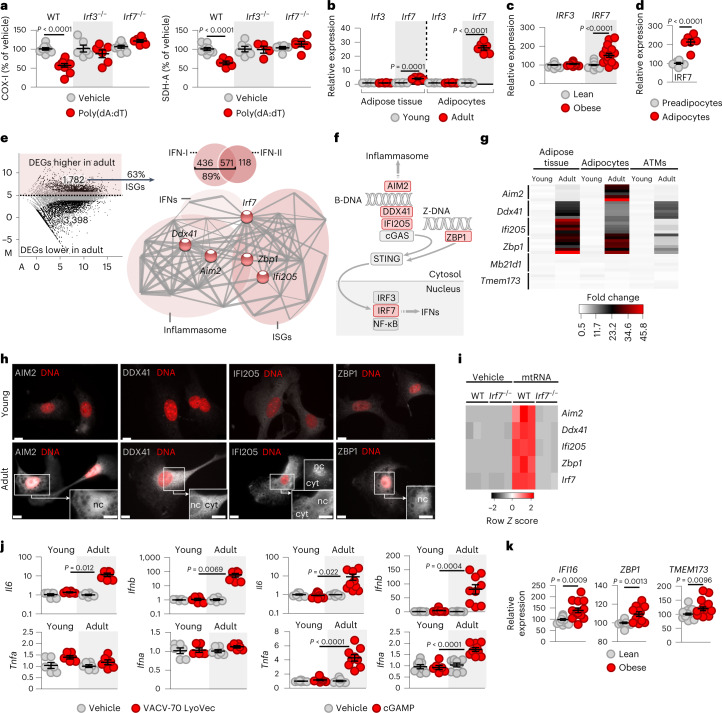
IRF7 is key for the IFN response to cytosolic mtRNA in adipocytes. **a**, Levels of COX-I and SDH-A in adipocytes following transfection with poly(dA:dT). *Irf3*^−/−^, adipocytes deficient in IRF3; *Irf7*^−/−^, adipocytes deficient in IRF7. **b**, Relative expression levels of *Irf3* and *Irf7* mRNA in inguinal adipose tissue and in adipocytes of young and adult mice. **c**, Expression levels of *IRF3* and *IRF7* mRNA in the adipose tissue of children, 0.3–6.9 years of age. **d**, IRF7 protein levels in human preadipocytes and hypertrophic adipocytes, shown in Fig. 1h. **e**, Volcano plot of differentially expressed genes (DEGs) of young and adult mouse subcutaneous adipose tissue. IFN-I, type I ISGs; IFN-II, type II ISGs. Protein–protein interaction network of differentially expressed genes over-represented in adult adipose tissue. IRF7 had a central position by interconnecting gene networks of IFNs, ISGs and inflammasome components. **f**, Signal pathways of IRF7-target genes. Gene products over-represented in adult mice are indicated in red. **g**, Transcription of IRF7-target genes in young adult mouse adipose tissue, adipocytes and adipose tissue macrophages (ATMs). *Mb21d1* is also known as *Cgas*, *Tmem173* as *Sting1*. **h**, Protein expression of IRF7-target genes in young and adult mouse adipocytes. cyt, cytosol; nc, nucleus; scale bar, 10 μm. **i**, Transcription of IRF7-target genes in mouse adipocytes transfected with mtRNA for 18 h. WT, wild-type; *Irf7*^−/−^, adipocytes deficient in IRF7. **j**, Response of young and adult mouse adipocytes to cytosolic DNA. Adipocytes were transfected with 1 μg ml^−1^ VACV-70, a ligand of IFI205, for 18 h or with 10 μg ml^−1^ cGAMP, a ligand of STING, for 2 h. **k**, Transcription of IRF7-target genes in subcutaneous adipose tissue in children, 0.3–6.9 years of age. Data are represented as mean ± s.e.m. *n* = 6 (**a**,**b**,**d**,**g**,**j**, left), *n* = 15 (**c**), *n* = 9 (**j**, right), *n* = 15 lean and *n* = 14 obese (**k**), *n* = 3 (**i**) biologically independent samples. The assay shown in **h** was repeated six times. Statistical significance was determined using Student’s two-tailed unpaired *t-*test and one-way ANOVA with Dunnett’s post-hoc test. Source data

Transcription of *Ifnb* is initiated by IRF3 and IRF7, which trigger the IFN response by forming homodimers or heterodimers^40,41^. Accordingly, the absence of adipocyte *Irf3* or *Irf7* was equally protective from mitochondrial damage in response to cytosolic poly(dA:dT) (Fig. 2a).

**Extended Data Fig. 2:**
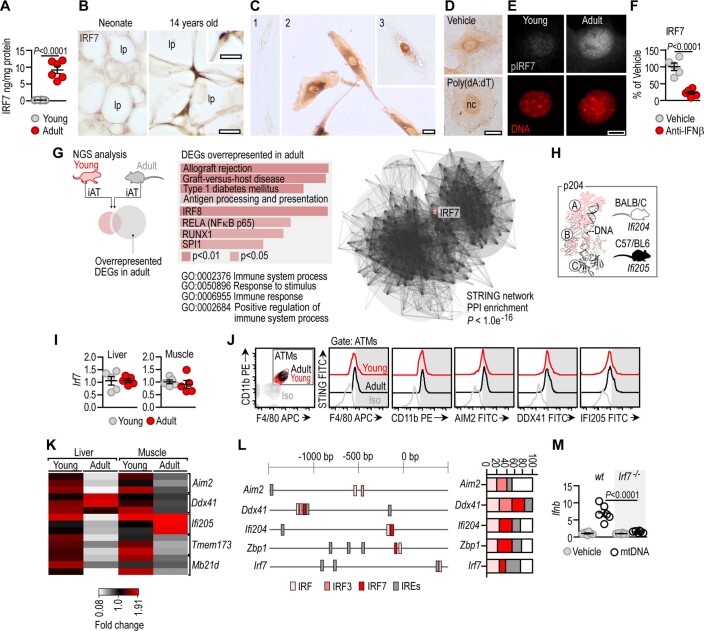
IRF7-controlled gene networks in mouse adipocytes. (**A**) Protein level of IRF7 in young and adult mouse adipocytes, determined with ELISA. N = 6 biologically independent samples. (**B**) Immunostaining of IRF7 in the subcutaneous adipose tissue of a human male neonate and a 14 years old male child. Scale 25 μm. lp: lipid droplet. Note the lack of staining in the infant sample, and the robust cytosolic staining in the 14 years old child. Insert shows nucleus associated IRF7 labeling. Assay repeated two times. (**C**) IRF7 immunostaining of *in vitro* cultured adipocytes of a human infant [1] and adolescents [2-3]. Note the lack of staining in the infant adipocytes. IRF7 was detectable in the cytoplasm [2] and rarely in the nuclei [3] of adipocytes from adolescents. Scale 50 μm. (**D**) IRF7 staining of murine adipocytes transfected with vehicle or 5 μg/ml poly(dA:dT) for 1 h. Note the enrichment of IRF7 in the nucleus of poly(dA:dT)-stimulated adipocytes, and indicative of IRF7 activation^41,92^. Scale 25 μm. Assay repeated six times. (**E**) Localization of Ser477-phosphorylated IRF7 in young and adult murine adipocytes, stimulated with 5 μg/ml poly(dA:dT) for 1 h. Scale 10 μm. (**F**) Level of IRF7 (determined with FACS analysis) in murine adipocytes treated with an antibody against IFNβ for 3 h. N = 5 biologically independent samples. (**G**) Scheme of NGS analysis. For RNA sequencing we obtained inguinal fat depots (iAT) of 3-3 mice at postnatal day 6 (young) and at week 8 (adult) and compared their transcriptional profiles. Gene ontology of differentially-expressed genes (DEGs) overrepresented in adults^93^. Protein-protein association network of DEGs overrepresented in adults. The position of IRF7 within the network is highlighted. (**H**) Structure of the DNA-sensor p204 (IFI204). The three DNA-binding domains are labeled A, B and C. p204 is encoded by *Ifi204* in BALB/C mice. In C57/BL6, however, *Ifi204* has a frameshift mutation and its function is taken over by *Ifi205* (refs. ^94–96^). In 3T3-L1 cells, which have a BALB/C origin, we measured *Ifi204*, whereas we measured *Ifi205* in adipocytes from C57/BL6 mice. (**I**) Level of *Irf7* in liver and quadriceps muscle of young and adult mice, N = 5 biologically independent samples. (**J**) FACS plot of ATMs from iAT. ATMs were defined as F4/80^+^, CD11b^+^. FACS analysis of IRF7-associated gene products of young and adult ATMs. Assay was repeated three times. Gating strategy is provided in ref. ^97^. (**K**) Expression of *Aim2, Ddx41, Ifi205, Tmem173* and *Mb21d1* in various organs. N = 3 biologically independent samples (**L**) Relative position and percentage of transcription factor binding sites in the promoters of IRF7-target genes. (**M**) Level of *Ifnb* mRNA in *wt* and *Irf7*^−*/*−^ adipocytes following mtRNA transfection, N = 6 biologically independent samples. Data are represented as mean ± SEM. Statistical significance was determined using Student’s 2-tailed unpaired *t-*test. Source data

We next measured transcription of *Irf3* and *Irf7* in adipocytes of young and adult mice. The transcript levels of *Irf3* were similar in young and adult adipocytes; however, the level of *Irf7* was magnitudes higher in adult adipocytes than in their young counterparts (Fig. 2b and Extended Data Fig. 2a). In adult adipocytes, IRF7 appeared in the cytoplasm and in the nucleus, and, upon stimulation, IRF7 translocated to the nucleus and become phosphorylated (Extended Data Fig. 2b–e). In children, the adipose tissue level of *IRF7* mRNA was strongly increased by overweight and obesity (body mass index standard deviation score over 1.28), while the level of *IRF3* mRNA was unrelated to obesity status (Fig. 2c). Accordingly, hypertrophic human adipocytes strongly expressed IRF7 protein (Fig. 2d), along with IFN-β (Fig. 1h). This is in agreement with previous findings suggesting that IRF3 is expressed constitutively, whereas IRF7 is an ISG^42^. The association of IRF7 expression with IFN-β^+^ hypertrophic adipocytes further supports this idea. Neutralizing IFN-β mitigated IRF7 protein expression in adipocytes in vitro (Extended Data Fig. 2f).

IRF7 plays a major role in the induction of the IFN response, even in the absence of IRF3. In turn, the IRF3-mediated IFN response remains minimal without the presence of even low amounts of IRF7 (ref. ^42^), underscoring the fact that IRF7 deficiency of young adipocytes is plausibly responsible for the lack of IFN response to mtRNA. Nevertheless, the importance of IRF7 in the regulation of IFN-β expression may depend on the immune context and the cell type^41,43^; thus we continued to define a possible role of IRF7 in the IFN response of adipocytes.

When we surveyed the transcriptional landscape of young and adult mouse inguinal adipocytes using next-generation sequencing (NGS), we found that an extensive, IRF7-associated gene network was strongly over-represented in adult adipocytes (Fig. 2e and Extended Data Fig. 2g). This gene network contained ISGs, genes encoding inflammasome components and IFNs (Fig. 2e). The most over-represented genes of this IRF7-associated network were *Aim2*, *Ddx41*, *Zbp1* and *Ifi205*, all of which are essential for the IFN response (Fig. 2f).

In brief, *Aim2* encodes the IFN-inducible protein absent in melanoma 2 (AIM2), which triggers cytosolic DNA inflammasome assembly^44^ (Fig. 2f). *Ddx41* encodes DEAD-box helicase 41 (DDX41), which recognizes cytosolic B-DNA and stimulates the IFN response (Fig. 2f). *Zbp1* encodes Z-DNA-binding protein 1 (ZBP1), also termed DAI^45^, which recognizes cytosolic Z-DNA (Fig. 2f), a prevalent form of DNA in the cytosol of transcriptionally active cells^46^. *Ifi205* encodes IFN-γ-inducible protein 205 (IFI205), the murine equivalent of the human IFI16 protein^44^, which recognizes cytosolic B-DNA and stimulates the IFN response (for details of the mouse strain-specific nomenclature of this protein, see Extended Data Fig. 2h)^40,41^.

The increase in the expression of this IRF7-associated gene network was confined to adipocytes (Fig. 2g) and was lacking in other cell types and organs that we tested (Extended Data Fig. 2i–k). Consistently, the AIM2, DDX41, IFI205 and ZBP1 proteins were minimally expressed in young adipocytes (Fig. 2h). In turn, AIM2, DDX41, IFI205 and ZBP1 were present in the perinuclear region and in the cytoplasm of adult adipocytes, coherent with their known role in monitoring specific subcellular compartments (Fig. 2h)^45,47^. Induced transcription of *Aim2*, *Ddx41*, *Zbp1*, *Ifi205* and *Irf7* was dependent on IRF7 (Fig. 2i), and coherently, the promoter regions of these genes contained IRF7-binding sequences (Extended Data Fig. 2l). The levels of IRF7-associated ISG-encoding transcripts, including *IFI16*, *ZBP1* and *TMEM173* (*STING1*), were increased in the adipose tissue of children with obesity (Fig. 2k), reflecting their elevated *IRF7* levels (Fig. 2c).

It is intriguing that the main function of this IRF7-associated gene network is to initiate the IFN response to cytosolic DNA, rather than to cytosolic dsRNA^48^. Consistently, cytosolic DNA failed to trigger an IFN response in young adipocytes (Fig. 2j) and in adipocytes lacking IRF7 expression (Extended Data Fig. 2m). As mtRNA efflux is inevitably associated with the escape of mtDNA to the cytosol^21^, this finding suggests that young adipocytes may be protected from an immune response to mtDNA, a known mechanism triggering obesity and obesity-induced metabolic diseases^31^.

Altogether, these findings indicate that young adipocytes are protected from an immune response to cytosolic mtRNA. This immune tolerant state was concomitant with the suppression of IRF7-controlled genes of the IFN response.

### Suppressed IRF7 signalling favours beige adipogenesis

**Fig. 3:**
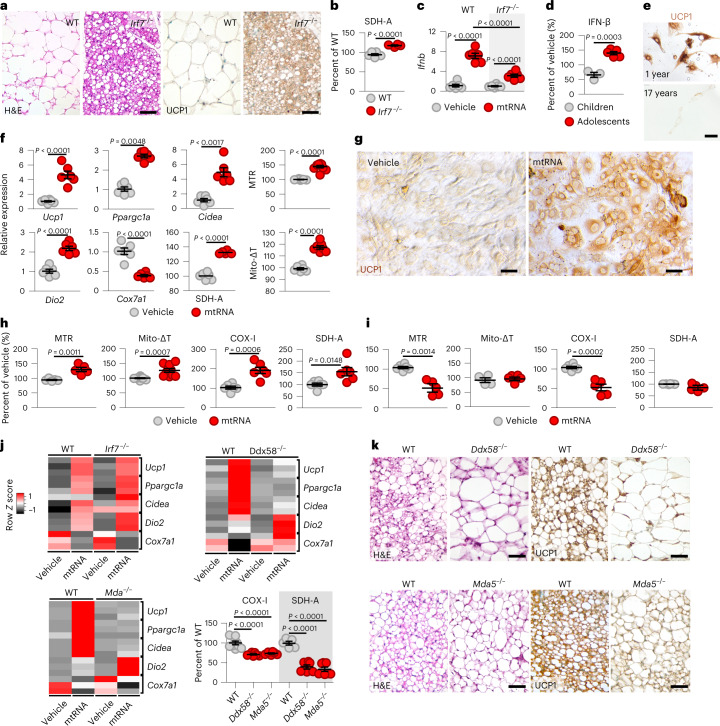
Cytosolic mtRNA induces beige adipocyte development. **a**, Histology of the inguinal adipose tissue of adult WT and IRF7-deficient *(Irf7*^−/−^*)* mice. UCP1, immunostaining of UCP1. Scale bar, 25 μm. **b**, Relative amount of the nuclear-encoded mitochondrial SDH-A in WT and *Irf7*^−/−^ adipocytes. **c**, *Ifnb* transcription of WT and *Irf7*^−/−^ adult adipocytes following transfection with vehicle or mtRNA (2 μg ml^−1^, 4 h). **d**, IFN-β protein expression of adipocytes from children (1–9 years) and adolescents (16–17 years), transfected with mtRNA. **e**, UCP1 immunostaining of adipocytes isolated from a 1-year-old and a 17-year-old donor. Scale bar, 50 μm. **f**, Transcription of mitobiogenesis and thermogenesis genes in young mouse adipocytes following transfection with vehicle or mtRNA (2 μg ml^−1^, 18 h). MTR, MTR fluorescence intensity, which is proportional to the amount of mitochondria. Mito-ΔT, mitochondrial temperature change, assessed with MitoThermoYellow staining. **g**, UCP1 immunostaining of mouse adipocytes transfected with vehicle or mtRNA for 18 h. Scale bar, 50 μm. **h**, Cytosolic delivery of 2 μg ml^−1^ mtRNA into adipocytes of human infants (1–2.5 years of age) and its effect on mitobiogenesis. MTR, MTR fluorescence intensity; Mito-ΔT, assessed with MitoThermoYellow staining; COX-I, COX-I activity; SDH-A, SDH-A activity. **i**, Cytosolic delivery of 2 μg ml^−1^ mtRNA into adipocytes of human adolescents (16–17 years of age), and its effect on mitobiogenesis. **j**, Heatmap showing expression levels of beige adipocyte genes in WT, *Irf7*^−/−^, RIG-I-deficient (*Ddx58*^−/−^) and MDA5-deficient (*Mda5*^−/−^) adipocytes transfected with vehicle or 2 μg ml^−1^ mtRNA for 18 h. Relative levels of mitochondrially encoded COX-I and nuclear-encoded SDH-A in young WT, *Ddx58*^−/−^ and *Mda5*^−/−^ adipocytes. Each data point represents adipocytes collected from five to seven mice. **k**, Histology of subcutaneous adipose tissue in young WT, *Ddx58*^−/−^ and *Mda5*^−/−^ mice. Note the absence of beige (multilocular) adipocytes in *Ddx58*^−*/*−^ and *Mda5*^−/−^ mice. Scale bar, 25 μm. Data are represented as mean ± s.e.m. *n* = 6 (**b**,**c**,**f**,**h** (COX-I and SDH-A levels), **j**), *n* = 5 (**d**,**h** (MTR and Mito-ΔT levels)) biologically independent samples. Assays shown in **a**,**e**,**g**,**k** were repeated six times. Heatmaps in **j** represent three replicates of independent analyses. Statistical significance was determined using Student’s two-tailed unpaired *t-*test (**b**–**d**,**f**,**h**) or one-way ANOVA with Dunnett’s post-hoc test (**j**). Source data

**Extended Data Fig. 3:**
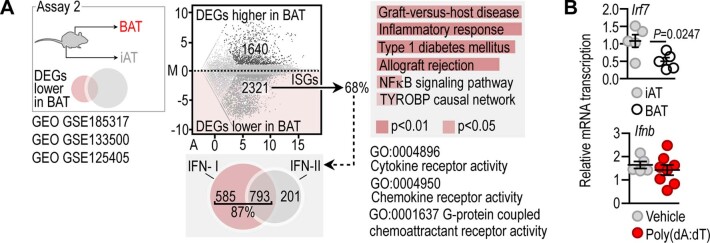
IRF7 expression in the brown adipose tissue in mouse. (**A**) Scheme of NGS analysis in adult mice. Replicate NGS analysis is available in ref. ^11^. iAT: inguinal adipose tissue, BAT: interscapular brown adipose tissue. DEGs underrepresented in BAT, and their gene ontology analysis (**B**) *Top:* Comparison of *Irf7* mRNA level in iAT and BAT. *Bottom: Ifnb* level in BAT transfected with vehicle of poly(dA:dT) for 18 h. N = 5 (*Top*) and N = 8 (*Bottom*) biologically independent samples. Source data

To further elucidate the role of IRF7 in adipocyte development, we analysed the subcutaneous adipose tissue morphology of mice lacking IRF7. In the absence of IRF7, mice had abundant beige adipocytes with strong uncoupling protein 1 (UCP1) expression, resembling the morphology of brown adipose tissue (Fig. 3a). Coherently, brown adipose tissue in adult mice was naturally deficient in *Irf7* and did not respond to cytosolic poly(dA:dT) with *Ifnb* expression (Extended Data Fig. 3a,b), in agreement with previous findings on a suppressed innate immune response in brown adipose tissue of mice^11,49,50^.

**Extended Data Fig. 4:**
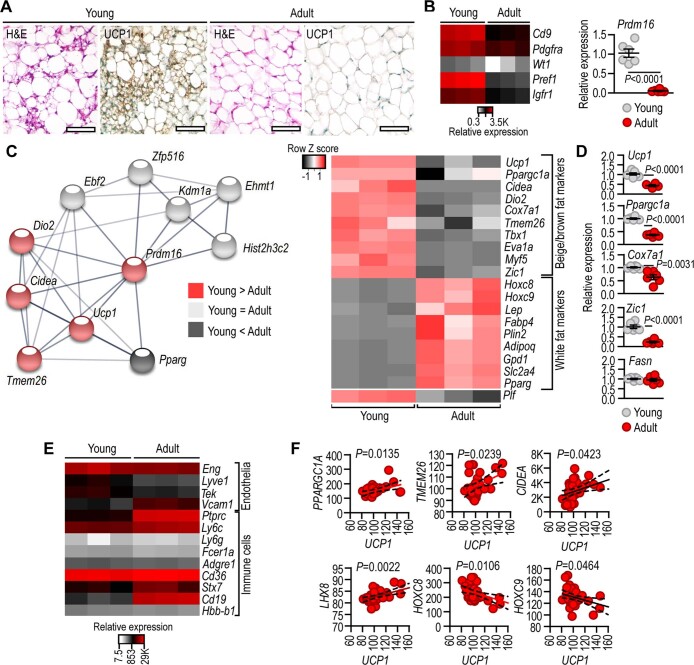
Characterization of young and adult adipose tissue. (**A**) Hematoxylin and eosin (H&E) staining, and immunostaining of UCP1 in mouse inguinal adipose tissue (iAT) of young and adult mice. Scale: 50 μm, also see further images of these samples in ref. ^11^. Assay was repeated six times for both age groups. (**B**) *Left:* Transcription of adipocyte progenitor genes in iAT. N = 3 biologically independent samples. *Pdgfra* and *Pref1* are associated with beige progenitors^98–101^; *Wt1* is a hallmark of epidydimal fat, and is lacking from iAT^102^. *Right:* Adipocyte transcription of *Prdm16*, a key regulator of thermogenic adipocyte development^28,78,103^. (**C**) Gene network associated with PRDM16 in young and adult mouse adipocytes. Red symbols indicate differentially expressed genes (DEGs) overrepresented in young adipocytes. Heat map summarizing the transcription level of beige/brown adipocyte marker genes and white adipocyte marker genes in young and adult iAT. N = 3 biologically independent samples. *Ucp1* is necessary for thermogenesis; *Ppargc1a* for mitochondrial biogenesis; *Cidea*, *Cox7a1, Dio2, Zic1* are associated with brown/beige adipocytes; *Tmem26* and *Tbx1* are beige adipocyte markers; *Eva1a* is a brown adipocyte marker^104–109^; *Myf5* is expressed by progenitors of brown adipocytes^80^. Levels of *Hoxc8* and *Hoxc9* increase along white adipocyte development^104^, although *Hoxc9* may also be a marker of beige adipocytes^109^. *Lep*, *Fabp4*, *Plin2*, *Adipoq*, *Gpd1*, *Slc2a4* and *Pparg* are associated with white adipocyte maturation^110^. *Plf* is expressed by small proliferating preadipocytes with a strong potential to develop into beige adipocytes^111^. (**D**) Expression levels of beige adipocyte genes and *Fasn* (encoding fatty acid synthase) in P6 and P56 adipocytes. N = 6 biologically independent samples. Data are represented as mean ± SEM. Statistical significance was determined using Student’s 2-tailed unpaired *t-*test. (**E**) Expression levels of endothelial and immune cell marker genes in young and adult iAT. N = 3 biologically independent samples (**F**) Correlation of *UCP1* levels with beige/brown adipocyte-associated transcripts (*PPARGC1A*, *TMEM26*, *CIDEA*, *LHX8*) and white adipocyte markers (*HOXC8*, *HOXC9*) in the subcutaneous adipose tissue of human male infants^12,104^, each data point represents one donor. *P*-values were determined with linear regression analysis. Age 0.2–3.5 years. Further details in refs. ^11,12,16,103,104^. Source data

Beige adipocytes are thermogenic; thus they help to dissipate energy stored in fat and eventually reduce body fat. The abundance of thermogenic adipocytes in IRF7-deficient mice is consistent with a previous finding that IRF7-deficient mice are protected from obesity^43^. IRF7-deficient adipocytes had increased expression of nuclear-encoded mitochondrial SDH-A (Fig. 3b), indicating increased mitobiogenesis. Moreover, adipocytes lacking IRF7 were protected from *Ifnb* expression triggered by cytosolic mtRNA (Fig. 3c). Of note, inguinal adipose tissue of young mice was rich in beige adipocytes, and adipocytes of young mice expressed signature genes of the thermogenic differentiation programme (Extended Data Fig. 4b–e). Adipocytes of lean children (1–4 years) were protected from IFN-β synthesis in response to cytosolic mtRNA and expressed UCP1, unlike adipocytes isolated from adolescents (16–17 years) (Fig. 3d,e and Extended Data Fig. 4f).

In summary, cytosolic mtRNA did not trigger IFN-β synthesis in young adipocytes. This trait was phenocopied by IRF7 deficiency and was associated with a thermogenic adipocyte phenotype. In mice, thermogenic fat cells were naturally deficient in IRF7, while, in humans, obesity increased the level of adipocyte IRF7.

Assuming that cytosolic mtRNA may play a role in the acquisition of the thermogenic phenotype, we next tested the effect of cytosolic mtRNA on the expression of genes required for mitochondrial thermogenesis and mitobiogenesis in young adipocytes. Cytosolic mtRNA triggered robust expression of *Ucp1* and genes associated with beige adipocytes (Fig. 3f): *Ppargc1a*, encoding the mitobiogenesis-stimulating peroxisome proliferator-activated receptor γ coactivator 1α; *Cidea*, encoding cell death-inducing DFFA-like effector A; and *Dio2*, encoding iodothyronine deiodinase 2. In young adipocytes, these responses occurred without induction of a robust immune response (Fig. 1c), and, ultimately, cytosolic mtRNA enhanced mitobiogenesis and the mitochondrial content of the adipocytes and stimulated mitochondrial thermogenesis (Fig. 3f,g). Similarly, cytosolic mtRNA increased mitobiogenesis, mitochondrial content and thermogenesis in adipocytes isolated from lean children (Fig. 3h), effects lacking in adipocytes of adolescents (Fig. 3i).

**Extended Data Fig. 5:**
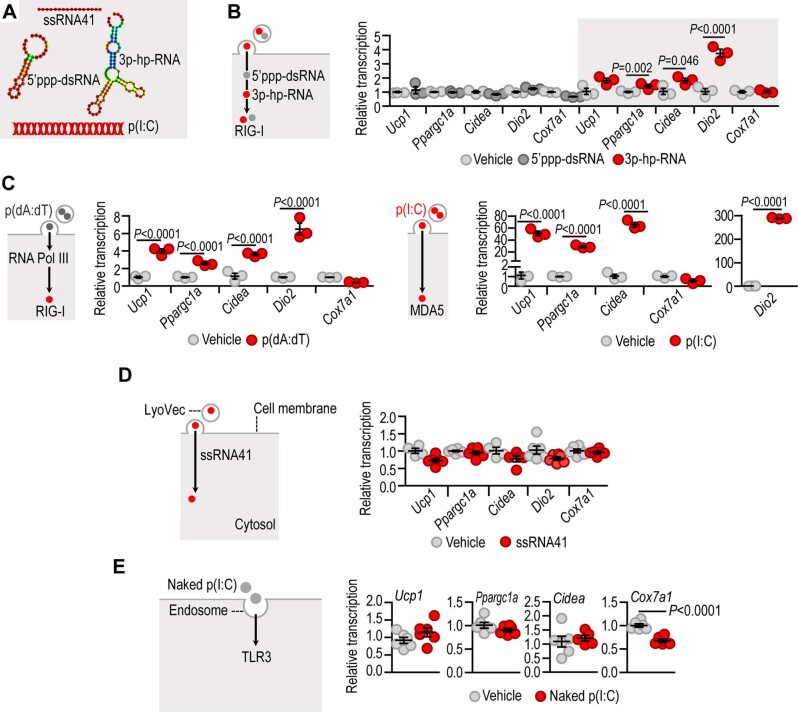
Effect of RIG-I and MDA5 ligands on beige adipocyte gene expression. (**A**) Secondary and schematic structures of the synthetic ligands used to activate cytosolic RNA sensors. ssRNA41: single-stranded RNA, 3p-hp-RNA: 5′ triphosphate hairpin RNA, is an RIG-I ligand^112^, 5′ppp-dsRNA: 5′ triphosphate dsRNA, a ligand for RIG-I, cytosolic p(I:C) activates MDA5 and RIG-I^34^, and cytosolic p(dA:dT) is transcribed into RNA and ultimately activates RIG-I^39^. (**B**,**C**) Adipocytes were transfected with RIG-I/MDA5 ligands: 5′ppp-dsRNA, 3p-hairpin-RNA, poly(dA:dT) and poly(I:C) in LyoVec. Levels of beige marker genes was measured 18 h after transfection. N = 3 biologically independent samples. (**D**) Adipocytes were transfected with 2 μg/ml ssRNA41 using the LyoVec transfection system for cytosol delivery. Levels of beige marker genes was measured 18 h after transfection. N = 6 biologically independent samples. (**E**) 3T3-L1 cells were treated with 5 μg/ml naked poly(I:C) to stimulate TLR3 and beige adipocyte gene transcription was then measured 18 h after treatment. N = 8 biologically independent samples. Data are represented as mean ± SEM. Statistical significance was determined using Student’s 2-tailed unpaired *t-*test. Source data

**Extended Data Fig. 6:**
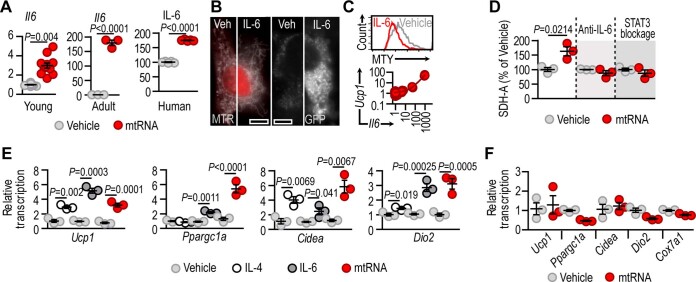
Effect of IL-6 on beige adipocyte gene expression. (**A**) *Left and Middle:* Expression of *Il6* mRNA in young and adult mouse adipocytes transfected with mtRNA for 18 h (N = 8, N = 3 biologically independent samples, respectively). *Right:* Level of IL-6 protein in mtRNA-transfected human adipocytes. N = 6 biologically independent samples. (**B**) Effect of 200 pg/ml IL-6 on the amount of newly synthesized (GFP-expressing) mitochondria in mouse adipocytes. Newly formed mitochondria were labeled with the BacMam-GFP transfection system. Scale: 50 μm. Assay was repeated three times. (**C**) Effect of 200 pg/ml IL-6 on the Mitothermo-Yellow (MTY) signal in 3T3-L1 cells. Assay was repeated three times. Correlation of *Il6* and *Ucp1* relative expression in mouse adipocytes, each data point represents a biologically independent sample. (**D**) SDH-A levels in adipocytes transfected with mtRNA. Anti-IL-6: cells were incubated with an antibody against IL-6. STAT-3 blockage: cells were treated with the JAK2/STAT3 inhibitor ruxolitinib. N = 3 biologically independent samples. (**E**) Transcription of beige adipocyte genes in mouse adipocytes treated with vehicle, 10 ng/ml IL-4, 200 pg/ml IL-6 or 2 μg/ml mtRNA for 18 h. N = 3 N = 6 biologically independent samples. (**F**) Expression of beige adipocyte genes in adipocytes treated with the JAK2/STAT3 inhibitor ruxolitinib as described^16^. N = 3 biologically independent samples. Data are represented as mean ± SEM. Statistical significance was determined using Student’s 2-tailed unpaired *t-*test. Source data

Cytosolic mtRNA may activate signalling through RIG-I and MDA5 (refs. ^33,34^). Coherently, synthetic activators of RIG-I and MDA5 triggered transcription of beige genes in adipocytes (Fig. 3j and Extended Data Fig. 5a–c), whereas cytosolic mtRNA was ineffective in inducing beige gene expression in adipocytes lacking RIG-I or MDA5 (Fig. 3j). Cytosolic single-stranded RNA or stimulation of cell membrane-associated Toll-like receptor 3 (TLR3) failed to mimic the effects of cytosolic mtRNA on beige gene transcription (Extended Data Fig. 5d,e). These findings show that only double-stranded, cytosolic RNA molecules can induce mitobiogenesis, plausibly via retrograde signalling from mitochondria to the nucleus. Provided the abundance of double-stranded motifs in mtRNA molecules, this finding further confirms the signalling role of mtRNA in the cytosol. RIG-I and MDA5 activation by mtRNA or synthetic ligands triggered *Il6* expression (Extended Data Fig. 6a), which is a known autocrine–paracrine signal of thermogenic adipocyte development^16,51,52^ (Extended Data Fig. 6b,c,e). Accordingly, blocking IL-6 signalling diminished the effect of mtRNA on beige adipogenesis (Extended Data Fig. 6d,f).

Notably, the absence of RIG-I or MDA5 compromised the expression of the nuclear-encoded mitochondrial SDH-A in adipocytes (Fig. 3j), suggesting that the mitochondrion-to-nucleus signalling role of mtRNA was lacking. Coherently, lack of RIG-I or MDA5 led to the loss of beige adipocytes in young mice (Fig. 3k).

Cytosolic mtRNA is, therefore, a strong inducer of beige features in young adipocytes by stimulating the expression of nuclear-encoded transcripts of mitobiogenesis and mitochondrial thermogenesis.

### Vitamin D suppresses IRF7 expression in young adipocytes

Young and thermogenic adipocytes were deficient in IRF7 and were protected from the adverse IFN-β-inducing effect of cytosolic mtRNA, enabling mtRNA to act as an intracellular signalling molecule. Repression of IRF7 may be thus key to promote the metabolically beneficial effects of mtRNA-mediated signalling in adipocytes. We next aimed to explore potential mechanisms that suppress the expression of IRF7 in young adipocytes.

**Fig. 4:**
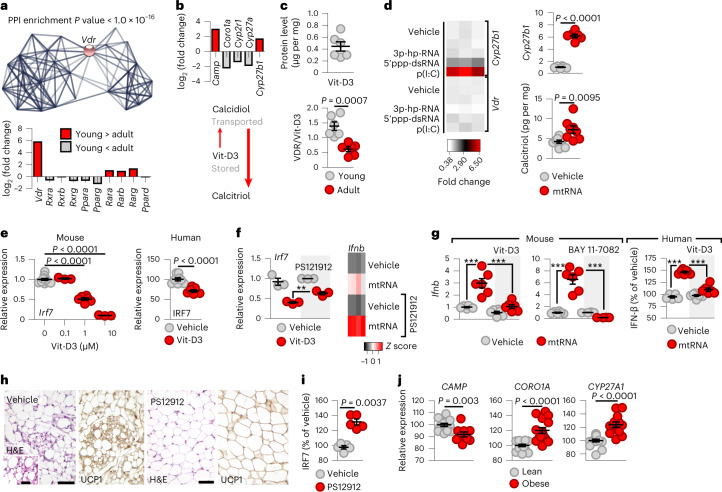
VDR diminishes IRF7 expression in young adipocytes. **a**, Genes over-represented in young mouse adipocytes belonged to the network associated with VDR signalling. Top, interactome map of gene products over-represented in young adipocytes. Bottom, comparison of young versus adult transcript levels of VDR and other nuclear receptors. PPI, protein–protein interaction network. **b**, Top, young versus adult transcript level of VDR-controlled genes and vitamin D metabolism genes. Bottom, scheme summarizing the dominant pathway of vitamin D metabolism in young mouse adipocytes. **c**, Level of Vit-D3 and the ratio of VDR/Vit-D3 in young mouse adipose tissue. **d**, Level of calcitriol-synthesizing *Cyp27b1* and *Vdr* in response to various cytosolic RNA species. 3p-hp-RNA, 5′ triphosphate hairpin RNA; 5′ppp-dsRNA, 5′ triphosphate double-stranded RNA; p(I:C), cytosolic polyinosinic:polycytidylic acid. Effect of cytosolic mtRNA on expression of *Cyp27b1* and Vit-D3–calcitriol converting activity (produced calcitriol per mg protein per min) of adipocytes transfected with vehicle or 2 μg ml^−1^ mtRNA for 18 h. **e**, Effect of 2 μg ml^−1^ cytosolic mtRNA on *Irf7* transcription in mouse adipocytes and IRF7 levels in human adipocytes. **f**, Effect of Vit-D3 on *Irf7* and *Ifnb* transcription in mouse adipocytes. mtRNA, transfection with 2 μg ml^−1^ mtRNA; PS121912, VDR inhibitor. **P = 0.0051. **g**, Response of mouse adipocytes pretreated with 1 μM Vit-D3 to cytosolic mtRNA. BAY 11-7082, NF-κB inhibitor. Response of human adipocytes treated with vehicle or Vit-D3 to cytosolic mtRNA. **h**, Histology of inguinal adipose tissue of young mice treated with vehicle or PS12912 for 5 d. Scale bar, 25 μm. **i**, Adipocyte IRF7 protein level of young mice treated with vehicle or PS12912 for 5 d. **j**, VDR-controlled gene expression in human subcutaneous adipose tissue, 0.3–6.9 years of age. Data are represented as mean ± s.e.m., and each data point represents a biological replicate. *n* = 6 (**c**); *n* = 3 in the heatmap, *n* = 6 for *Cyp27b1* level and *n* = 8 for calcitriol level (**d**); *n* = 6 mice and *n* = 8 humans (**e**); *n* = 3 and 4 (**f**); *n* = 6 (**g**); *n* = 5 (**i**); *n* = 13 lean and *n* = 11 obese patients (**j**). The assay shown in **h** was repeated six times. Statistical significance was determined using Student’s two-tailed unpaired *t*-test (**d**,**e**, right; **f**,**g**,**i**,**j**) or one-way ANOVA with Dunnett’s post-hoc test (**e**, left). Source data

Our NGS analysis revealed prominent expression of VDR-controlled gene networks in young adipocytes (Fig. 4a), and VDR is known to suppress cytosolic RNA-induced IRF7 expression^53,54^ and the IFN response^55,56^. The transcriptional activity of VDR was confirmed by the pattern of VDR-controlled gene expression in young adipocytes (Fig. 4b). For example, the known VDR target *Camp*, encoding the adipose tissue-enriched antimicrobial peptide cathelicidin^57^, was highly expressed in young adipocytes (Fig. 4b). By contrast, the VDR-repressed gene *Coro1a* had a low transcript level in young adipocytes (Fig. 4b). *Coro1a* encodes coronin A1, also known as tryptophan–aspartate-containing coat protein^58^. Foetal adipose tissue accumulates vitamin D before birth^59^, and, accordingly, the transcription of vitamin D-metabolizing enzymes favoured the storage of vitamin D3 (Vit-D3) and the synthesis of the potent VDR agonist calcitriol in young adipocytes (Fig. 4b,c).

Adipose tissue of young mice was rich in Vit-D3, and VDR protein expression was higher in young adipose tissue than in its adult counterpart (Fig. 4c). Moreover, cytosolic dsRNA and mtRNA increased transcription of the calcitriol-synthesis gene *Cyp27b1* and enhanced Vit-D3–calcitriol conversion in adipocytes (Fig. 4d). Vit-D3 effectively suppressed *Irf7* transcription in mouse and human adipocytes (Fig. 4e). This effect was VDR dependent, and inhibition of VDR signalling augmented mtRNA-induced *Ifnb* transcription (Fig. 4f). In turn, Vit-D3 abrogated *Ifnb* expression in mouse adipocytes in response to cytosolic mtRNA (Fig. 4g). This effect of Vit-D3 mimicked that of a potent nuclear factor (NF)-κB inhibitor (Fig. 4g). Similarly, Vit-D3 mitigated IFN-β production in human adult adipocytes in response to cytosolic mtRNA (Fig. 4g).

### Obesity in early postnatal life triggers adipocyte IRF7 expression

**Fig. 5:**
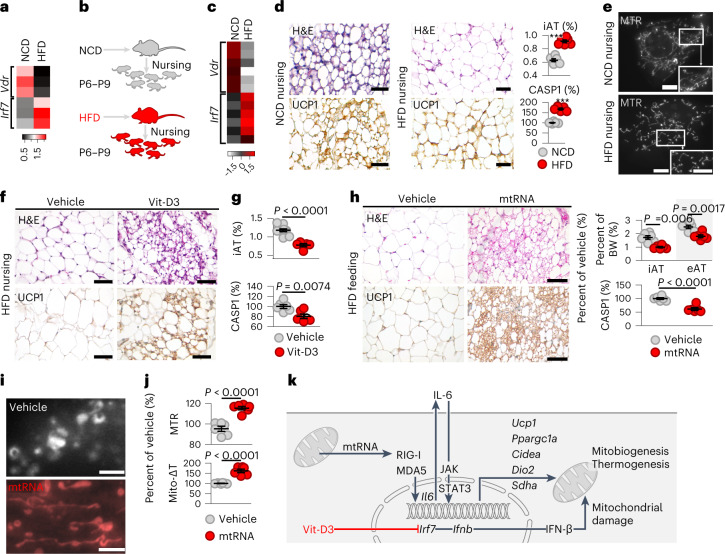
Effect of cytosolic mtRNA combined with Vit-D3 treatment on diet-induced obesity. **a**, Levels of *Vdr* and *Irf7* in inguinal adipose tissue of HFD-fed adult mice. NCD, normal chow diet. **b**, Nursing mice received an HFD or an NCD between postnatal day (P6) and postnatal day 9 (P9) of the offspring. Mice nursed by NCD-fed or HFD-fed dams were analysed at postnatal day 10. **c**, *Vdr* and *Irf7* expression in the inguinal adipose tissue of the offspring. **d**, Left, histology of inguinal adipose tissue (iAT). UCP1, UCP1 immunostaining; scale bar, 50 μm. Note the lack of multilocular adipocytes in mice nursed by HFD-fed dams. Right, ratio of inguinal adipose tissue and body weight, and inflammasome-associated caspase 1 (CASP1) activity of adipocytes. **e**, Mitochondrial network in adipocytes of the offspring. Scale bar, 10 μm. **f**, Mice were nursed by HFD-fed dams and treated with vehicle or Vit-D3 from postnatal day 6 to postnatal day 9. Histology of inguinal adipose tissue on postnatal day 10. Scale bar, 50 μm. **g**, Ratio of inguinal adipose tissue and body weight, and CASP1 activity of adipocytes. **h**, In adult HFD-fed mice, inguinal adipose tissue was transfected with vehicle or 0.6 μg per g body weight (BW) mtRNA for 14 d. Both groups received 4 ng per g body weight Vit-D3 daily. Histology of inguinal adipose tissue of vehicle- or mtRNA-transfected mice. Adipose tissue weight/body weight ratio and CASP1 activity of adipocytes. eAT, epididymal adipose tissue. **i**, Mitochondrial network of adipocytes isolated from vehicle- or mtRNA-transfected mice. Scale bar, 10 μm. Note the expansion of the mitochondrial network after mtRNA treatment. **j**, Mitochondrial mass (relative MTR fluorescent intensity) and Mito-ΔT in adipocytes isolated from vehicle- or mtRNA-transfected mice. Data are represented as mean ± s.e.m. *n* = 6 (**a**–**d**,**g**,**h**,**j**) biologically independent samples. The assay shown in **d**,**e**,**f**,**h**,**i** was repeated six times. Statistical significance was determined using Student’s two-tailed unpaired *t-*test. **k**, Scheme of retrograde mitochondrion-to-nucleus signalling through mtRNA. Cytosolic mtRNA activates IL-6 synthesis, an inducer of thermogenic fat cell differentiation in the newborn, through RIG-I and MDA5. VDR simultaneously suppresses IRF7 expression and abrogates the IFN response to cytosolic mtRNA. The net effect of mtRNA is mitobiogenesis and thermogenesis. Source data

Inhibition of VDR signalling in young mice led to the loss of beige adipocytes in adipose tissue (Fig. 4h) and increased IRF7 protein levels in adipocytes (Fig. 4i). Paediatric obesity was associated with compromised expression of VDR-controlled gene networks in the adipose tissue of children and lower expression of *CYP27A1*, which is involved in the initial activation of Vit-D3 (Fig. 4j). These alterations were associated with an increased *IRF7* level (Fig. 2c) and the expression of IRF7-target genes (Fig. 4j). Similarly, diet-induced obesity increased the adipose tissue level of *Irf7* and abrogated *Vdr* expression in mice (Fig. 5a). Altogether, these data show that obesity is linked to deficient VDR signalling, which is further associated with increased IRF7 expression.

We next studied a mouse model of childhood obesity using newborn mice nursed by dams fed a high-fat diet (HFD) (Fig. 5b)^60^. Adipocytes of the offspring of HFD-fed dams had compromised *Vdr* expression and robust *Irf7* expression (Fig. 5c), and beige adipocytes were lacking from their subcutaneous adipose tissue (Fig. 5d). Ultimately, obesity developed in offspring, and adipocytes showed inflammasome activation, a hallmark of adipose tissue inflammation^61^ (Fig. 5d). Moreover, the mitochondrial network was compromised in adipocytes of young mice nursed by HFD-fed dams (Fig. 5e). In turn, Vit-D3 treatment reverted these adverse changes and protected beige adipocyte content in young mice (Fig. 5f), alleviating obesity and adipocyte inflammation (Fig. 5g).

**Extended Data Fig. 7:**
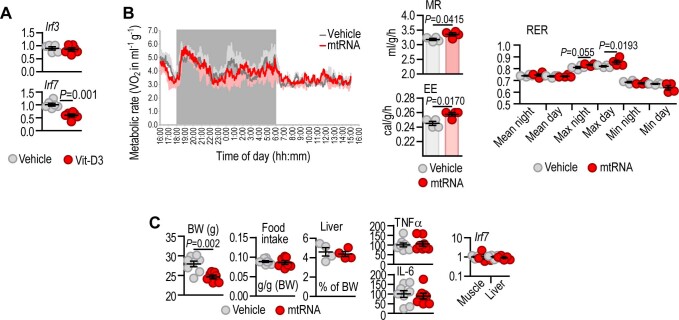
Metabolic role of mtRNA-mediated signaling. (**A**) Level of *Irf3* and *Irf7* in mouse adipocytes treated with 1 μM Vit-D3 for 18 h. N = 6 biologically independent sample. (**B**) Indirect calorimetry assay of high-fat diet (HFD)-fed adult male C57BL/6 mice, N = 6 biologically independent samples. The inguinal fat depot was transfected with vehicle or with 0.6 μg/g body weight (BW) per day mtRNA for 14 days. The mtRNA was delivered into the adipocyte cytoplasm using magnetofection. Both groups received 4 ng/g BW Vit-D3 daily. MR: metabolic rate, EE: energy expenditure, RER: respiratory exchange rate (**C**) BW, daily food intake normalized to BW (N = 8 biologically independent samples), and liver weight normalized to BW (N = 4 biologically independent samples). Plasma level of TNFα and IL-6 (% of vehicle) from vehicle- or mtRNA-transfected mice, and the level of *Irf7* in quadriceps muscle and liver (N = 8 biologically independent samples). Data are represented as mean ± SEM. Statistical significance was determined using Student’s 2-tailed unpaired *t-*test. Source data

**Extended Data Fig. 8:**
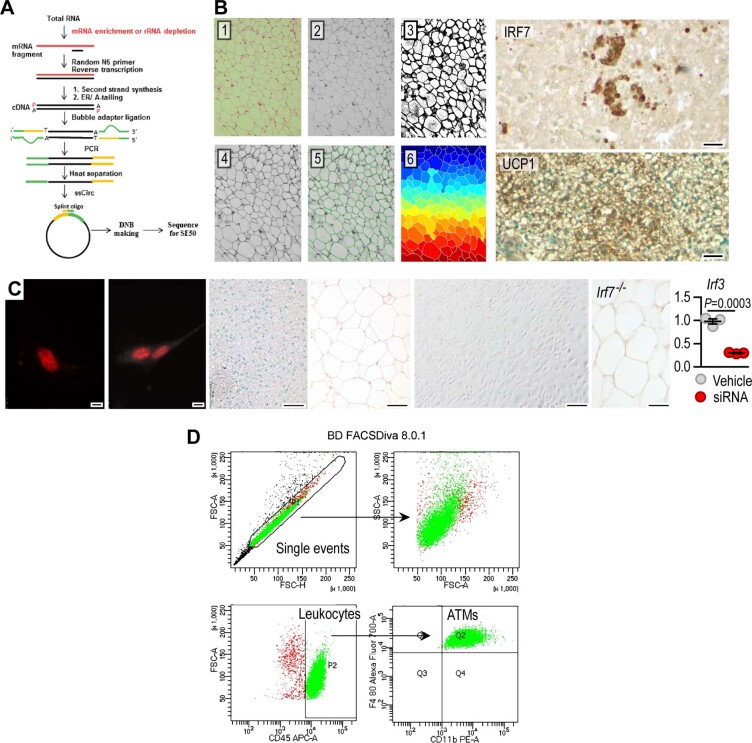
Technical information on next-generation sequencing and image analysis. (**A**) Workflow of the next-generation sequencing analysis. (**B**) Left: Steps of image analysis in histomorphometry [1-6]. Right: Positive controls in immunohistochemistry. Newborn liver was used for testing IRF7 antibody. Note the hematopoietic cell foci. Brown adipose tissue of mouse was used as a control for UCP1 antibody. Methyl-green counterstaining for nuclei. Further validation provided in ref. ^11^. Scale 50 μm. Performed three times. (**C**) Negative control specimens. *Left:* Adipocytes *in vitro*, stained with secondary antibodies only; nuclei are labeled with DAPI. Scale: 10 μm. *Middle:* Mouse brown adipose tissue section labeled with secondary antibody only. Human adipose tissue labeled with secondary antibody only. Scale: 20 μm. *Right*: Adipose tissue of an IRF7-deficient mouse labeled with an antibody against IRF7. *Irf3* level in 3T3-L1 cells transfected with *Irf3* siRNA for 48 h. (**D**) Example of adipose tissue macrophage (ATM) gating strategy. Further details in refs. ^97^ and ^113^. Source data

In adult HFD-fed mice, cytosolic delivery of mtRNA into the inguinal adipose tissue depot, combined with Vit-D3 treatment, reduced *Irf7* levels (and not *Irf3* levels) and increased beige adipocyte content (Fig. 5h and Extended Data Fig. 7a), alleviated obesity and adipocyte inflammation and increased mitochondrial mass, thermogenesis and energy expenditure (Fig. 5h–j and Extended Data Figs. 7b,c and 8).

## Discussion

**Fig. 6:**
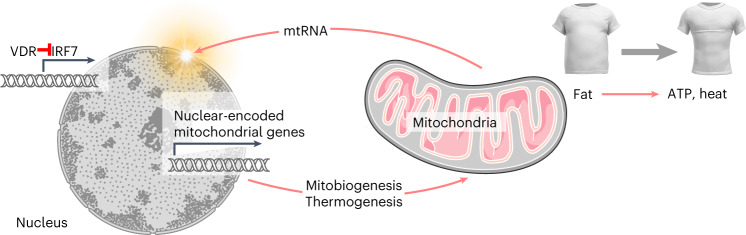
Role of mtRNA signalling in young adipocytes. Albeit cytosolic mtRNA is a harmful signal, it can act as a metabolically beneficial mitochondrion-to-nucleus messenger when IRF7 expression is suppressed. VDR is an effective suppressor of IRF7 and abrogates the IFN response to cytosolic mtRNA in young adipocytes. Young adipocytes are hence immune tolerant sites for mitochondria, allowing retrograde mitochondrion-to-nucleus signalling through mtRNA, which is key for mitobiogenesis and beige fat development. See also a Video Summary available in the Supplementary Information.

Overall, our findings show that, in young adipocytes, cytosolic mtRNA stimulates expression of nuclear-encoded mitochondrial genes and promotes beige adipocyte development through the RIG-I–MDA5–interleukin (IL)-6–signal transducer and activator of transcription (STAT)3 pathway (Fig. 5k). This mitochondrion-to-nucleus signalling is effective when the immune response against cytosolic mtRNA is suppressed by VDR activation and consequently low IRF7 expression in adipocytes (Fig. 5k). These mechanisms protect against obesity by evoking thermogenic potential in adipocytes and promoting thermogenesis from stored fat (Fig. 6, Video Summary in the Supplementary Information).

Adipose tissue inflammation is considered deleterious for metabolism^4,62^; however, multiple lines of evidence implicate IL-6–Janus kinase (JAK)–STAT3 signalling in the differentiation of thermogenic adipose tissue^16,51,52,63,64^, and an autocrine IL-6–STAT3 signalling loop is sustained by breast milk-derived lipids in the adipose tissue of newborns^16^. Several inflammatory signalling mechanisms that trigger obesity-associated metabolic impairment also sustain beige adipocytes^35,52,64,65^. Here we report the unexpected finding that beige adipocyte development is promoted by a potentially inflammation-evoking cytosolic RNA signal released by the mitochondria of adipocytes.

The endosymbiotic origin of mitochondria has led to a metabolic co-dependence of mitochondria and host cells^22^. This is driven by bidirectional signalling between the nucleus and the mitochondria, as the majority of genes required for the maintenance of mitochondria are encoded in the nuclear genome. We show that, analogous to a symbiont–host interaction, efflux of mtRNA from the mitochondria activates cytosolic RNA sensors that stimulate an autocrine IL-6–STAT3 signalling loop, ultimately triggering nuclear expression of beige adipocyte genes. Noncoding RNA species of mitochondria are known to increase transcription of mitochondrial genome-encoded genes^66^, and noncoding RNA signals are thought to function as mitochondrion-to-nucleus signals^67^. As a comparable mechanism, we show that mtRNA species boost transcription of nuclear genome-encoded genes for mitobiogenesis and thermogenesis. This is key for mitobiogenesis as the necessary proteins are encoded in the nuclear genome^22^.

RIG-I and MDA5 are sensors of cytosolic mtRNA. RIG-I detects dsRNA species with or without a 5′-triphosphate end, MDA5 binds uncapped RNA, and RIG-I and MDA5 selectively recognize short and long dsRNA species, respectively^33,34^. Given the prokaryote origin of mitochondria, various mtRNA species such as mitochondrial ribosomal RNA, uncapped mitochondrial mRNA and noncoding mtRNA can potentially stimulate the cytosolic RNA-sensor system^24,33,34^. We found that beige adipocyte gene transcription was stimulated by RIG-I activation using cytosolic poly(dA:dT) and also by MDA5 activation using cytosolic high-molecular-weight poly(I:C) but not with cytosolic single-stranded RNA. Coherently, deficiency in RIG-I and MDA5 signalling in mice compromised mtRNA-mediated beige adipocyte development, abrogated nucleus-encoded SDH-A expression and mitobiogenesis and promoted the loss of beige adipocytes. These findings are in agreement with a recent report showing that mice lacking RIG-I are prone to obesity and obesity-associated insulin resistance^68^, despite being protected from the IFN response^68^.

However, the excess release of mitochondrial content activates an IFN response, which is detrimental for thermogenic fat development^28,31,69,70^, increases mitochondrial permeability^71^, augments inflammasome activation and pyroptosis^72^, triggers obesity, mitochondrial dysfunction and the mitochondrial pathway of adipocyte apoptosis and may aggravate obesity-associated metabolic diseases^29,30,73,74^. We show here that young adipocytes have suppressed the IFN response to cytosolic mtRNA due to their suppressed IRF7 expression. As IRF7 is an IFN-inducible gene product, this is in agreement with previous findings that suggest that beige adipocytes repress IFN signalling^28^ and the expression of ISGs in adipocytes promotes obesity and adipose tissue inflammation^29^. IRF3 and IRF7 are key transcription factors regulating ISGs; however, their role in obesity development is conflicting. Lack of IRF7 protects from diet-induced obesity^43^, while IRF3-deficient mice develop obesity spontaneously^75^. We found that *Irf3* expression was similar in adipocytes of young and adult mice, while *Irf7* was minimally expressed in young adipocytes. Similarly, paediatric obesity was associated with increased *IRF7* expression in adipose tissue, without a significant increase in *IRF3* levels. Diet-induced obesity in mice and obesity in children were associated with robust expression of genes encoding IRF7-associated pathways. This is in agreement with findings on increased expression of the IRF7-target genes *AIM2* and *IFI16* in type 2 diabetic obese human adipose tissue^76^. Adipose tissue expansion and obesity are associated with the activation of STAT1 and NF-κB signalling, which may account for the increase in IRF7 expression during postnatal adipocyte development and in obesity^29,64,77^.

We also show that suppressed *Irf7* expression and mtRNA-induced *Ifnb* expression were associated with the thermogenic adipocyte phenotype. Inguinal adipocytes of young mice and interscapular brown adipose tissue of adult mice were deficient in IRF7 and were lacking mtRNA-induced *Ifnb* expression. Interscapular brown adipocytes are strongly thermogenic cells and are descendants of the Myf5^+^ lineage derived from skeletal muscle progenitors in mice^78–80^. Primates and humans have different thermoregulatory mechanisms than those of small rodents^17^, and thermogenic adipocytes of a newborn human are scattered within white fat depots^12^. Equivalents of these cells appear in newborn mice as well^11^. These thermogenic fat cells are unrelated to Myf5^+^ progenitors, and they develop from progenitors of white fat cells^79^. It appears that thermogenic fat cells in newborn subcutaneous tissue and in adult interscapular adipose tissue have distinct transcriptional profiles and specific developmental programmes^11,81^. Nevertheless, this study shows that, irrespective of their origin and development, thermogenic adipocytes have suppressed IRF7 expression and are eventually lacking an IFN response to cytosolic mtRNA. The IFN response augments inflammasome activation, which further damages mitochondria^71^. However, a gene network encoding inflammasome proteins had suppressed expression in young (thermogenic) adipocytes, suggesting their potential protection from mitochondrion-damaging inflammasome activation. These, in our understanding, favour the expansion of the mitochondrial network and allow mitochondrial thermogenesis.

We show that VDR signalling contributes to the suppression of IRF7 expression in adipocytes and that cytosolic mtRNA stimulates calcitriol synthesis and hence supplies a VDR ligand in young adipocytes. However, as a limitation of this study, we have not explored further the mechanism of *Irf7* suppression in interscapular brown adipose tissue in mice, leaving open the possibility that this unique thermogenic fat depot has a VDR-independent mechanism that suppresses *Irf7* expression.

VDR signalling is involved in the innate immune response in adipose tissue^57^, and VDR activation inhibits the inflammasome and the IFN response^53–56,82^. Vit-D3 supplementation is now routine in postnatal care; however, Vit-D3 deficiency is prevalent among obese children and adolescents and is a risk factor for metabolic diseases^83,84^. Vit-D3–VDR signalling is proposed to inhibit weight gain by activating UCP3 in the muscle^85^, although VDR overexpression promotes weight gain in mice^86^. Indeed, the promotion of formula feeding originally served to increase the supply of Vit-D3 and induce weight gain^87^, but formula lacks the maternal lipid species that maintain beige fat and has obesogenic effects^16^. VDR signalling was impaired in the adipose tissue of obese children; therefore, despite increasing Vit-D3 levels, formula milk is not sufficient to trigger beige adipogenesis. However, when Vit-D3 supplementation is combined with stimulation of cytosolic mtRNA signalling, beige adipocytes develop and obesity is reduced.

In summary, the thermogenic potential of young adipocytes in early postnatal life is dependent on mtRNA-mediated signalling and suppression of the immune response to cytosolic mtRNA. In obesity, adipocytes respond with inflammation to mtRNA, which is unfavourable for the mitochondrial network. Repressing this immune response along with restoring mtRNA-mediated mitochondrion-to-nucleus signalling may represent an effective mechanism to increase beige fat and mitigate obesity.

## Methods

### Animals and cells

We used WT male C57BL/6 (Charles River Laboratories), *Irf7*^−/−^ (Riken), *Ddx58*^−***/***−^ and *Mda5*^−/−^ (kindly provided by G. Hartmann, University of Bonn, Germany) mice. Mice were housed under SPF conditions. Animal experiments were approved by local ethics committees. Primary mouse adipocytes were isolated by collagenase digestion and separation of cell fractions and subsequently analysed or cultured, as described previously^16^.

### Human samples

Subcutaneous adipose tissue (groin region, ischiorectal fossa, abdominal and pectoral fat depots) from human infants, adolescents and young adults was collected in the Leipzig Childhood Adipose Tissue cohort and at the University of Debrecen during elective surgery^2^. For all children included in the study, written informed consent was obtained from parents or guardians, and the study has been conducted in accordance with ethical guidelines of the Declaration of Helsinki. The study protocol was approved by the local ethics committee of the Medical Faculty, University of Leipzig (265-08-ff, NCT02208141) and the University of Debrecen (RKEB 6057 and 6149). Adult adipocyte samples were collected in our previous study^16^. In Fig. 3h,i, we used adipocytes obtained from the groin region.

### mRNA analysis and next-generation sequencing

Extraction of total RNA was performed as described previously^11^. qPCR assays were carried out on the Quantabio platform, using *Bactin* (*Actb*), *Gapdh* and *Ppia* as references. Primer sequences are provided in Supplementary Table 1. NGS analysis was carried out on the BGISEQ-500 platform by BGI Genomics, generating about 26.20 million reads per sample (Extended Data Fig. 8). EnrichR, P1nther and Interferome 2.0 were used for annotation of transcripts; clustered image maps (CIMs, heatmaps) were rendered by CIMMiner and Heatmapper. Gene expression in human samples was quantified by Illumina HT-12 v4 Gene Expression BeadChip arrays, and data were background corrected and quantile normalized^11^.

### Supplementary methods

Cytosolic delivery of mtRNA, ELISA assays, flow cytometry, histology, image analysis and TEM analysis are provided in the Supplementary Information and Extended Data Fig. 8.

### Data representation and statistics

Data are represented as mean ± s.e.m., along with each individual data point. When data are represented as CIMs to visualize gene transcription differences between experimental conditions, we indicate fold changes or *Z* scores of the relative abundance. Statistical tests and significance are indicated in the respective figures.

### Reporting summary

Further information on research design is available in the Nature Portfolio Reporting Summary linked to this article.

### Supplementary information

Supplementary Information Supplementary Tables 1 and 2 and Methods.

Reporting Summary

Supplementary Video Video abstract.

## Data Availability

Data are available for secondary use upon request, and key experimental data are accessible via Figshare (10.6084/m9.figshare.21202400). FlowRepository identifiers of flow cytometry data are as follows: FR-FCM-Z236, FR-FCM-Z2R6, FR-FCM-ZYPU, FR-FCM-ZYUU, FR-FCM-Z5QA. NGS data are deposited at GEO under the accession number GSE185317. For secondary analysis, we used our previously published NGS datasets, with accession numbers GSE125405 and GSE133500. Source data are provided with this paper.

## Source data

Source Data Fig. 1 Source data for graphs/statistics, histology images and other source data.
Source Data Fig. 2 Source data for graphs/statistics, histology images and other source data.
Source Data Fig. 3 Source data for graphs/statistics, histology images and other source data.
Source Data Fig. 4 Source data for graphs/statistics, histology images and other source data.
Source Data Fig. 5 Source data for graphs/statistics, histology images and other source data.
Source Data Extended Data Fig. 1 Source data for graphs/statistics, histology images and other source data.
Source Data Extended Data Fig. 2 Source data for graphs/statistics, histology images and other source data.
Source Data Extended Data Fig. 3 Source data for graphs/statistics.
Source Data Extended Data Fig. 4 Source data for graphs/statistics, histology images and other source data.
Source Data Extended Data Fig. 5 Source data for graphs/statistics.
Source Data Extended Data Fig. 6 Source data for graphs/statistics, histology images and other source data.
Source Data Extended Data Fig. 7 Source data for graphs/statistics and other source data.
Source Data Extended Data Fig. 8 Source data for graphs/statistics and histology images.
