# Supplementary material for: Mitochondrial RNA stimulates beige adipocyte development in young mice
Source: Nat Metab. 2022 Nov 28;4(12):1684–96. doi: 10.1038/s42255-022-00683-w (PMC9771821; doi:10.1038/s42255-022-00683-w)
Supplement: Supplementary file 1 — Supplementary Tables 1 and 2 and Methods. [file 42255_2022_683_MOESM1_ESM.pdf]

nature metabolism

Article https://doi.org/10.1038/s42255-022-00683-w

Mitochondrial RNA stimulates beige adipocyte development in young mice

In the format provided by the authors and unedited

Supplementary Table 1. Mouse qPCR primer sequences used in the study (Merck)

Bactin	fw	GCACCAGGGTGTGATGGTG
	rev	CCAGATCTTCTCCATGTCGTCC
Ppia	fw	ATTTCTTTTGACTTGCGGGC
	rev	AGACTTGAAGGGGAATG
Gapdh	fw	TGACGTGCCGCCTGGAGAAA
	rev	AGTGTAGCCCAAGATGCCCTTCAG
Aim2	fw	GATTCAAAGTGCAGGTGCGG
	rev	TCTGAGGGTTAGCTTGAGGAC
Ddx41	fw	ACAGGAGAAGCGGTTGCCTTTC
	rev	GACGGCAGTAATACTCCAGGATG
Ifi204	fw	CAGGGAAAATGGAAGTGGTG
	rev	CAGAGAGGTTCTCCCGACTG
Zbp1	fw	AACCCTCAATCAAGTCCTTTACCGC
	rev	TCTTCCACGTCTGTCCGTCATAGCT
Mb21d	fw	AGGAAGCCCTGCTGTAACACTTCT
	rev	AGCCAGCCTTGAATAGGTAGGTAGTCCT
Tmem173	fw	GGGCCCTGTCACTTTTGGTC
	rev	GAGTATGGCATCAGCAGCCAC
Irf3	fw	GGCTTGTGATGGTCAAGGTT
	rev	CATGTCCTCCACCAAGTCCT
Irf7	fw	CGACTTCAGCACTTTCTTCCGAGA
	rev	AGATGGTGTAGTGTGGTGACCCTT
Il6	fw	GCTACCAAACTGGATATAATCAGGA
	rev	CCAGGTAGCTATGGTACTCCAGAA
Tnfa	fw	TGCCTATGTCTCAGCCTCTTC
	rev	GAGGCCATTTGGGAACTTCT
Ifnb	fw	CCAGCTCCAAGAAAGGACGA
	rev	CGCCCTGTAGGTGAGGTTGAT
Ifna	fw	TGAAGGACAGGAAGGACTTTG
	rev	GAATGAGTCTAGGAGGGTTGT
Rn16s	fw	ACACCGGAATGCCTAAAGGA
	rev	ATACCGCGGCCGTTAAACTT
Nd1	fw	GCTTTACGAGCCGTAGCCCA
	rev	GGGTCAGGCTGGCAGAAGTAA
Co3	fw	CCAAGGCCACCACACTCCTA
	rev	GGTCAGCAGCCTCCTAGATCA
Ucp1	fw	CCTGCCTCTCTCGGAAACAA
	rev	CTGTAGGCTGCCCAATGAAC
Ppargc1	fw	GACTCAGTGTCACCACCGAAA
	rev	TGAACGAGAGCGCATCCTT
Cox7a1	fw	ATGAGGGCCCTACGGGTCTC
	rev	CATTGTCGGCCTGGAAGAG
Cidea	fw	TACTACCCGGTGTCCATTTCT
	rev	ATCACAACTGGCCTGGTTACG
Dio2	fw	GTCCGCAAATGACCCCTTT
	rev	CCCACCCACTCTCTGACTTTC

Ifi205	fw	CAAGCAGGCCACTTCTGTTG
	rev	TCAAACGGGTCTGTTGCAGT
Ddx58	fw	CAAACCGGGCAACAGGAATG
	rev	ATCTCCGCTGGCTCTGAATG
Zic1	fw	TTTCCTGGCTGCGGCAAGGTTT
	rev	ACGTGCATGTGCTTCTTGCGGT
Fasn	fw	GGAGGTGGTGATAGCCGGTAT
	rev	TGGGTAATCCATAGAGCCCAG
Prdm16	fw	ATCCACAGCACGGTGAAGCCAT
	rev	ACATCTGCCCACAGTCCTTGCA
Mda5	fw	GTCTTCTGCCTGCTGTCGAT
	rev	CAAACTTGTGTCTGATTCTGTGGT
Cyp27b1	fw	AGCTCCTGCGACAAGAAAGT
	rev	ATTCTTCACCATCCGCCGTTA
Vdr	fw	ACTTTGACCGGAATGTGCCT
	rev	CATGCTCCGCCTGAAGAAAC

Supplementary Table 1. (cont.) Primers for measuring mouse mtDNA copy number

Nd1	fw	GCTTTACGAGCCGTAGCCCA
	rev	GGGTCAGGCTGGCAGAAGTAA
16S	fw	ACACCGGAATGCCTAAAGGA
	rev	ATACCGCGGCCGTTAAACTT
HK2	fw	GCCAGCCTCTCCTGATTTTAGTGT
	rev	GGGAACACAAAAGACCTCTTCTGG

Supplementary Table 2. Antibodies used in the study (h, human; m, mouse)

**Target**	**Cat. No.**	**IgG type, source**
h/m STING	NBP2-24683	Rabbit polyclonal, 1:250<br>Novus Biologicals, Denver, CO
	13657	Rabbit monoclonal, 1:250<br>Cell Signaling Technology, Danvers, MA
h/m AIM2	201708-T10	Rabbit polyclonal, 1:125<br>Sino Biological, Eschborn, Germany
h/m DDX41	102459-T32	Rabbit polyclonal, 1:50<br>Sino Biological, Eschborn, Germany
h/m p204 (IFI16)	NBP2-27153	Rabbit Polyclonal, 1:100<br>Novus Biologicals, Denver, CO
h/m ZBP1	207744-T08	Rabbit polyclonal, 1:150<br>Sino Biological, Eschborn, Germany
h/m LC3	L8918	Rabbit polyclonal, 1:500, Merck Sigma-Aldrich<br>St. Louis, MO
h/m UCP1	PA1-24894	Rabbit polyclonal, 1:200<br>ThermoFisher Scientific, Rockford, IL
h/m DDX41	102459-T32	Rabbit polyclonal, 1:150<br>Invitrogen, Carlsbad, CA
J2 (dsRNA)	Anti-dsRNA [J2]	Mouse monoclonal, 1:100, clone J2<br>Absolute Antibody, Wilton, UK
m IRF7<br>h/m IRF7	12-5829-82<br>PA5-20280	PE-conjugated monoclonal IgG, clone MNGPKL,<br>and matching isotype IgG, 1:500, ThermoFisher,<br>Waltham, MA

h/m Ser477 p-IRF7	PA5106184	Rabbit polyclonal, 1:150<br>Invitrogen, Carlsbad, CA<br>Rabbit polyclonal, 1:250<br>Invitrogen, Carlsbad, CA
m F4/80 antigen<br>m CD11b	sc-377009<br><br>E-AB-F1081E	F4/80 APC, IL-6 PE, CD11b APC or PE (clone M1/70, FACS analysis, 1:500), eBioscience, ThermoFisher, Waltham, MA, Santa Cruz Biotech (for IHC, 1:500)
m cGAS	79978	Rabbit monoclonal, 1:200, clone E5V3W<br>Cell Signaling Technology, Danvers, MA
h/m IFNβ	PA5-20390<br>BMS1044FI	Rabbit polyclonal, 1:100 (neutralization assay)<br>Mouse monoclonal (clone A1), 1:500<br>Invitrogen, Carlsbad, CA
Rabbit anti-goat IgG	F-2765	H+L, cross-adsorbed, FITC, polyclonal, secondary antibody, 1:500, Invitrogen, Carlsbad, CA
Goat anti-rabbit IgG	A16096	Goat anti-rabbit IgG (H+L), 1:500, HRP-conjugated<br>Invitrogen, Carlsbad, CA

Supplementary Methods

Activation and inhibition of cytosolic DNA/RNA sensors

To stimulate RIG-I/MDA5, we transfected adipocytes at 80% confluency with high molecular weight polyinosine-polycytidylic acid (p(I:C)) or poly(deoxyadenylic-deoxythymidylic) acid (p(dA:dT)) using the LyoVec cationic lipid-based transfection reagent (InvivoGene, Toulouse, France). Control cells were treated with LyoVec transfection reagent only. We used 0.5-5 µg/ml p(dA:dT) or p(I:C), and cells were analyzed 2–24 h after transfection. IFI16/p204/IFI205 was activated with 1 µg/ml VACV-70 conjugated to LyoVec transfection reagent (InvivoGene; 18 h) [1]. To activate STING, we treated adipocytes with cGAMP (InvivoGene, Toulouse, France) for 18 h. Treatments are summarized in the table below. *Irf3* expression was suppressed by siRNA silencing, using oligos from Thermo Fisher (Silencer™ Pre-Designed siRNA, Cat. No.: AM16708, siRNA ID: 184585).

**Activation of cytosolic nucleic acid sensors with various ligands**			
Receptor	**Ligand**	**EC_{50}**	**Applied concentration**
**STING**	2´3 cGAMP	20 nM	10 µg/ml
**cGAS**	poly(dA:dT)	40-200 ng/ml	0.5-5 µg/ml
	human/mouse mtDNA	-	2 µg/ml
	pCMV6 circular DNA	-	1 µg/well
**RIG-I**	3p-hpRNA	5 ng/ml	0.5 µg/ml
	5´ppp-dsRNA	1.2 nM	1 µg/ml
	poly(I:C) HMW	70±10 ng/ml	0.5 µg/ml
**RIG-I** and **MDA5**	poly(dA:dT) 18 h	40-200 ng/ml	2.5-5 µg/ml
	low molecular weight poly(I:C)	82±8 ng/ml	1 µg/ml
	human/mouse mtRNA	-	2 µg/well
**AIM2**	poly(dA:dT)	40-200 ng/ml	2.5-5 µg/ml
**DDX41**	poly(dA:dT)	40-200 ng/ml	2.5-5 µg/ml
	dsDNA (VACV-70)	-	1 µg/ml
**IFI16 (p204** or **IFI205)**	poly(dA:dT)	40-200 ng/ml	2.5-5 µg/ml
	dsDNA (VACV-70)	-	1 µg/ml
**ZBP1**	poly(dA:dT)	40-200 ng/ml	2.5-5 µg/ml

TLR3 was stimulated with naked p(I:C) (Sigma-Aldrich, 10 ng/ml, 18 h). As negative control we used ssRNA (InvivoGene). NFκB was inhibited with 5 μM BAY 11-7082 and JAK2/STAT3 with 280 nM ruxolitinib (Cayman Chemical Company, Ann Arbor, MI).

Vit-D3 and calcitriol were purchased from Sigma-Aldrich; IFNβ, IL-4, IL-6 and from ImmunoTools (Friesoythe, Germany). Isoproterenol and fumonisin B1 were purchased from Sigma-Aldrich and from Cayman Chemical Company, respectively. To test the inhibitory effect of Vit-D3 on IRF7 signaling, adipocytes were treated with 1 μM Vit-D3 for 48 h, and were transfected with mtRNA for 18 h. VDR was inhibited with PS121912, as described [2]. Cellular uptake of cGAMP is dependent on the transporter Slc19a1 [3], the level of which was similar in P6 and P56 adipocytes (GEO submission #GSE154925).

ELISA assays

Tissue samples were weighed and homogenized in RIPA buffer using a Roche bead mill homogenizer at 6,500 rpm for 1 min. Cell culture supernatants and plasma samples were centrifuged at 0.8 g for 10 min to remove cell debris, and supernatants were used for analysis. We used commercial ELISA kits to measure the levels of IL-6, TNFα (Fisher Scientific), IRF7, Vit-D3, calcitriol and VDR (MBS1605109, MBS268259, MBS288322, MBS2701844, MyBioSource). All samples were stored at -80°C until analysis.

mtRNA isolation and *in vitro* transfection

Adipocyte mitochondria were isolated with a commercial mitochondrial isolation kit (Thermo Fisher Scientific, Waltham, MA), using mouse adipocytes or human THP1 cells. Mitochondrial RNA (mtRNA) was isolated by lysing the mitochondrial pellet with TRI Reagent (Sigma-Aldrich), as described [4]. Adipocytes were transfected with 2 µg of mtRNA in 6- or 24-well plates with cells at 80–90% confluency. As a transfection reagent we used Lipofectamine 3000 (Invitrogen) at a 1:3 ratio. Control cells received transfection reagent only. Cells were analyzed 18 h after transfection.

mtDNA isolation and transfection

Mitochondrial DNA (mtDNA) was isolated from mitochondria pellets using TRI Reagent (Merck Sigma-Aldrich) and reconstituted in TE buffer (10 mM Tris-HCL, 1 mM EDTA, pH 8.0). Agarose gel electrophoresis was used to examine mtDNA integrity. Adipocytes were transfected for 18 h with 1-2 µg/ml mtDNA using the TurboFect Transfection Reagent. Control cells received transfection reagent only.

Cytosolic mtRNA and mtDNA isolation

Cytosol fractions of adipocytes were collected by subcellular fractionation of the cytoplasm and the cell organelles using digitonin, as described [5,6]. Digitonin buffer contained 150 mM NaCl, 50 mM HEPES (pH 7.4) and 25 µg/ml digitonin (D141, Merck Sigma-Aldrich). Treated cells were processed until the step in which cytosol was obtained as described [4]. Cytosolic fractions (250 µl) were added to 750 µl TRI Reagent (T3934, Merck Sigma-Aldrich) and total DNA and RNA extraction was performed as described [7]. Isolated RNA was reverse transcribed into cDNA, *Bactin* and *Ppia* were used as reference genes. DNA was reconstituted in TE buffer and adjusted to 10 ng/µl. We performed qPCR using HK2 as a

reference nuclear genome-encoded gene, and measured the DNA copy number of mtDNA-encoded genes. We calculated the copy number according to the formula:

$$\Delta Ct = Ct_{Target\ gene} - Ct_{Reference\ gene} \quad (1)$$
$$\text{mtDNA copy number} = 2X2^{(\Delta Ct)}$$

Histology, immunofluorescence and image analysis

Tissues were fixed with 4% paraformaldehyde and embedded in paraffin, as described (1). Sections were stained with hematoxylin and eosin (Carl Roth, Karlsruhe, Germany). Antibodies are listed in Supplementary Table 2. UCP1, IFI16, AIM2 immunohistochemistry was performed on paraffin-embedded tissue sections. For histomorphometry of fat cells we used Image J, with an image-processing algorithm that incorporated the Euclidean distance-based Watershed transformation to segment the images. Briefly, binarized images were generated using Otsu's method for thresholding; enhanced images were generated using contrast limited adaptive histogram equalization (CLAHE), and finally segmented images were generated using the Watershed transformation (Extended Data Figure 8). For fluorescent microscopy of STING, cGAS, AIM2, DDX41, IFI205 and ZBP1 murine or human preadipocytes were grown on optical transparent glass-bottom plates (Greiner Bio-One GmbH, Frickenhausen, Germany) or glass coverslips, and labeled with antibodies listed in Supplementary Table 2. Negative control specimens of our fluorescent imaging and immunostaining are shown in Extended Data Figure 8. Histology images were adjusted to equal white balance after acquisition. Mitochondrial content and morphology was analyzed with ImageJ, as described [4]. Beige adipose area was measured with our custom-developed image analysis software (BeAR© v1.0, [4]).

Quantification of UCP1 staining

In vitro UCP1 immunostaining was performed in 6-well culture plates, and samples were imaged and the optical density was measured using digital image analysis. Original images are available upon request through Figshare. Mitochondria were also labeled using an SDH-A histochemistry assay (BioOptica).

Adipocyte differentiation

Mouse or human preadipocytes of the stromal vascular fraction (SVF) were isolated and maintained as described [7-9]. To ensure the depletion of adipose tissue macrophages (ATMs) from the harvested preadipocytes, we used magnetic bead cell purification of the SVF with an antibody against the F4/80 antigen (Miltenyi Biotec, Bergisch Gladbach, Germany) [10]. Human subcutaneous adipose tissue preadipocytes were harvested as described [7,8]. Preadipocytes were maintained in cell culture medium supplemented with 20 μg/ml insulin. To induce white differentiation of preadipocytes, we treated the cells with 50 μM IBMX, 1 μM dexamethasone, 1 μM rosiglitazone and 20 μg/ml insulin (all from Merck Sigma-Aldrich), as described [4]. Lipid content was labeled with Oil red O.

Flow cytometry analysis of DNA sensors, mitochondrial biogenesis, mitochondrial content and mitochondrial uncoupling

Mitochondrial content was analyzed with MitoTracker dyes (Thermo Fisher Scientific). Mitochondrial biogenesis was detected with the MitoBiogenesis™ Flow Cytometry Kit

(Abcam, Cambridge, UK). MitoThermo Yellow (MTY), a temperature-sensitive fluorescent probe [11], was used to assess mitochondrial thermogenesis and uncoupling, as described [12,13]. Temperature difference between the control and the test groups was expressed as Mito-ΔT, and shown in the respective figures. MTY was developed and provided by Dr. Y-T. Chang (Center for Self-Assembly and Complexity, Institute for Basic Science & Department of Chemistry, Pohang University of Science and Technology, Pohang 37673, Republic of Korea). We used MTY for FACS analysis at 0.1 ng/ml to label 10^6/ml cells. Cells were maintained at 37°C throughout the assay. DNA sensors (STING, p204, AIM2, DDX41) were detected with unconjugated antibodies (listed in Supplementary Table 2) and labeled with an FITC-conjugated secondary antibody for FACS analysis. Nucleic acids were labeled with Sytox Green (Thermo Fisher). Flow Repository identifiers of raw FACS data are as follows: #FR-FCM-Z236, #FR-FCM-Z2R6, #FR-FCM-ZYPU, #FR-FCM-ZYUU, #FR-FCM-Z5QA.

Imaging of mitochondrial content

For fluorescent microscopy of mitochondrial content and morphology, preadipocytes were grown on optical transparent glass-bottom plates (Greiner Bio-One GmbH, Frickenhausen, Germany) or glass coverslips. Functional mitochondria were labeled with MitoTracker Red. Mitochondria were also labeled with GFP using the BacMam 2.0 transfection system (Fisher Scientific). Mitochondrial respiration was evaluated with the WST-81 assay (Carl Roth), as described [14]. SDH-A and COX-I level was measured with flow cytometry and spectrophotometry (Abcam MitoBiogenesis Kits). Inflammasome activity was measured with the Caspase-Glo 1 Inflammasome Assay (Promega Co., Madison, WI).

High-fat diet feeding and indirect calorimetry

Respiratory exchange rate (RER), oxygen consumption (VO_2) and energy expenditure (EE) were measured in each individual mouse for 24 h using a small animal indirect calorimetry system (CaloBox, Phenosys, Germany). Mean RER, VO_2 and EE values were determined over 7 h in the middle of both the day and the night phases. Basal glucose levels and glucose tolerance were measured as described [7]. For HFD-feeding of mice (dams with litters P6 to P9, or mice at P28 for 12 weeks) we used a rodent HFD from SSNIFF Spezialdiäten (Soest, Germany, E15725-347) [7]. Vit-D3 was supplemented in diet, mtRNA was transfected in the iAT with magnetofection for 14 days. As a normal chow diet we used rodent diet from SSNIFF Spezialdiäten (Soest, Germany, E15051-047).

Magnetofection of mtRNA

In vivo delivery of mtRNA into the cytosol of adipocytes was achieved with magnetofection, using mtRNA–magnetic nanoparticle complexes (DogtorMag, OzBiosciences, San Diego, CA). Briefly, 15 μg mtRNA-nanoparticle complexes were injected into the iAT of mice, and enrichment of the magnetic nanoparticles was ensured by magnetic exposure of the fat depot, as described [15].

Cell viability assay

We used the Presto Blue Cell Viability Assay (Thermo Fisher Scientific) and the Rotitest Vital (Carl Roth) assays.

Institutional Review Board Statement

Research involving animals was approved by the regional governmental ethics and animal welfare committee in Tübingen, Germany (#1511; #1557; #1492; #1546; #o.232-1,2,4,5).

Software

We used the following software for data collection and analysis: QuantStudio™ Design & Analysis Software, Thermofisher Scientific v1.5.0 (for qPCR); Leica Application Suite X Leica 3.7.2.22383 (for microscopy of immunofluorescence); Olympus CellSense Entry Olympus 2.3 (for histology imaging and cell culture imaging); Leica Application Suite Z Leica 3.4.0 (for histology imaging); AUTOsoft Autobio Co. LTD 2.6.9 (for ELISA); Fusion FX6 Edge Vilber 18.02 and NanoDrop 2000/2000c Thermofisher Scentific 1.6.198 (for RNA quantity); RNAfold WebServer (University of Vienna, 2.4.18, for RNA structure prediction); ImageJ, Open Source, 1.52v (for histomorphometry), Motic Images Plus 3.0 V137 (for histology imaging), Adobe Photoshop and Adobe Illustrator (for figure assembly).

Acknowledgements for the supplemental information

The VDR inhibitor was provided by Prof. Dr. Leggy A. Arnold, University of Wisconsin, USA. MTY was developed and provided by Dr. Y-T. Chang (Center for Self-assembly and Complexity, Institute for Basic Science & Department of Chemistry, Pohang University of Science and Technology, Republic of Korea. The authors thank Prof. Hartmut Geiger (Ulm University) for providing access to the FACS equipment. The assistance of Katharina Schormair and Burak Yildiz in image analysis is much appreciated. The contribution of Vincent Pflüger, Yun Chen, Antonia Stubenvoll, Angelika Bauer is acknowledged. Elements of the 3D artwork used in the graphical abstract and the video summary was provided by Dreamstime Stock Photography.

References

1 Unterholzner, L. *et al.* IFI16 is an innate immune sensor for intracellular DNA. *Nature immunology* **11**, 997-1004, doi:10.1038/ni.1932 (2010).

2 Sidhu, P. S. *et al.* Anticancer activity of VDR-coregulator inhibitor PS121912. *Cancer Chemother Pharmacol* **74**, 787-798, doi:10.1007/s00280-014-2549-y (2014).

3 Ritchie, C., Cordova, A. F., Hess, G. T., Bassik, M. C. & Li, L. SLC19A1 Is an Importer of the Immunotransmitter cGAMP. *Molecular Cell* **75**, 372-381.e375, doi:10.1016/j.molcel.2019.05.006 (2019).

4 Yu, H. *et al.* Breast milk alkylglycerols sustain beige adipocytes through adipose tissue macrophages. *The Journal of Clinical Investigation* **129**, 2485-2499, doi:10.1172/JCI125646 (2019).

5 Holden, P. & Horton, W. A. Crude subcellular fractionation of cultured mammalian cell lines. *BMC research notes* **2**, 243, doi:10.1186/1756-0500-2-243 (2009).

6 Dixit, B., Vanhoozer, S., Anti, N. A., O'Connor, M. S. & Boominathan, A. Rapid enrichment of mitochondria from mammalian cell cultures using digitonin. *MethodsX* **8**, 101197, doi:https://doi.org/10.1016/j.mex.2020.101197 (2021).

7 Waqas, S. F. H. *et al.* Neuropeptide FF increases M2 activation and self-renewal of adipose tissue macrophages. *The Journal of Clinical Investigation* **127**, 2842-2854 (2017).

8 Waqas, S. F. H. *et al.* Adipose tissue macrophages develop from bone marrow-independent progenitors in *Xenopus laevis* and mouse. *Journal of Leukocyte Biology* **102**, 845-855 (2017).

9 Hausman, D. B., Park, H. J. & Hausman, G. J. Isolation and culture of preadipocytes from rodent white adipose tissue. *Methods in Molecular Biology* **456**, 201-219, doi:10.1007/978-1-59745-245-8_15 (2008).

10 Ampem, G. & Röszer, T. in *Nuclear Receptors: Methods and Experimental Protocols* (ed Mostafa Z. Badr) 225-236 (Springer New York, 2019).

11 Arai, S. *et al.* Mitochondria-targeted fluorescent thermometer monitors intracellular temperature gradient. *Chemical Communications* **51**, 8044-8047, doi:10.1039/c5cc01088h (2015).

12 Lane, N. Hot mitochondria? *PLOS Biology* **16**, e2005113, doi:10.1371/journal.pbio.2005113 (2018).

13 Chrétien, D. *et al.* Mitochondria are physiologically maintained at close to 50 °C. *PLOS Biology* **16**, e2003992, doi:10.1371/journal.pbio.2003992 (2018).

14 Karabatsiakis, A. *et al.* Mitochondrial respiration in peripheral blood mononuclear cells correlates with depressive subsymptoms and severity of major depression. *Translational Psychiatry* **4**, e397-e397, doi:10.1038/tp.2014.44 (2014).

15 Plank, C. & Rosenecker, J. Magnetofection: The Use of Magnetic Nanoparticles for Nucleic Acid Delivery. *Cold Spring Harbor Protocols* **2009**, pdb.prot5230, doi:10.1101/pdb.prot5230 (2009).
