# Supplementary material for: Mitochondrial RNA stimulates beige adipocyte development in young mice
Source: Nat Metab. 2022 Nov 28;4(12):1684–96. doi: 10.1038/s42255-022-00683-w (PMC9771821; doi:10.1038/s42255-022-00683-w)
Supplement: Supplementary file 2 — Reporting Summary [file 42255_2022_683_MOESM2_ESM.pdf]

nature portfolio

Corresponding author(s): Tamás Röszer

Last updated by author(s): Sep 27, 2022

Reporting Summary

Nature Portfolio wishes to improve the reproducibility of the work that we publish. This form provides structure for consistency and transparency in reporting. For further information on Nature Portfolio policies, see our Editorial Policies and the Editorial Policy Checklist.

Statistics

For all statistical analyses, confirm that the following items are present in the figure legend, table legend, main text, or Methods section.

n/a	Confirmed	
☐	☒	The exact sample size (n) for each experimental group/condition, given as a discrete number and unit of measurement
☐	☒	A statement on whether measurements were taken from distinct samples or whether the same sample was measured repeatedly
☐	☒	The statistical test(s) used AND whether they are one- or two-sided<br>*Only common tests should be described solely by name; describe more complex techniques in the Methods section.*
☒	☐	A description of all covariates tested
☐	☒	A description of any assumptions or corrections, such as tests of normality and adjustment for multiple comparisons
☐	☒	A full description of the statistical parameters including central tendency (e.g. means) or other basic estimates (e.g. regression coefficient) AND variation (e.g. standard deviation) or associated estimates of uncertainty (e.g. confidence intervals)
☐	☒	For null hypothesis testing, the test statistic (e.g. F, t, r) with confidence intervals, effect sizes, degrees of freedom and P value noted<br>*Give P values as exact values whenever suitable.*
☒	☐	For Bayesian analysis, information on the choice of priors and Markov chain Monte Carlo settings
☒	☐	For hierarchical and complex designs, identification of the appropriate level for tests and full reporting of outcomes
☒	☐	Estimates of effect sizes (e.g. Cohen's d, Pearson's r), indicating how they were calculated

Our web collection on statistics for biologists contains articles on many of the points above.

Software and code

Policy information about availability of computer code

Data collection	QuantStudio™ Design & Analysis Software, Thermofisher Scientific v1.5.0 (for qPCR); Leica Application Suite X Leica 3.7.2.22383 (for microscopy of immunofluorescence); Olympus CellSense Entry Olympus 2.3 (for histology imaging and cell culture imaging); Leica Application Suite Z Leica 3.4.0 (for histology imaging); Motic Images Plus 3.0 V137 (for histology imaging); AUTOsoft Autobio Co. LTD 2.6.9 (for ELISA); Fusion FX6 Edge Vilber 18.02 and NanoDrop 2000/2000c Thermofisher Scentific 1.6.198 (for RNA quantity)
Data analysis	Workflow described in the Supplementary Information. We used Excel, GraphPad Prism, FlowJo, ImageJ for data analysis.

For manuscripts utilizing custom algorithms or software that are central to the research but not yet described in published literature, software must be made available to editors and reviewers. We strongly encourage code deposition in a community repository (e.g. GitHub). See the Nature Portfolio guidelines for submitting code & software for further information.

Data

Policy information about availability of data

All manuscripts must include a data availability statement. This statement should provide the following information, where applicable:

- Accession codes, unique identifiers, or web links for publicly available datasets
- A description of any restrictions on data availability
- For clinical datasets or third party data, please ensure that the statement adheres to our policy

Data are available for secondary use upon request, and key experimental data are accessible via Figshare. Flow Repository identifiers of FACS data are as follows: #FR-FCM-Z236, #FR-FCM-Z2R6, #FR-FCM-ZYPU, #FR-FCM-ZYUU, FR-FCM-Z5QA. NGS data are deposited at GEO with the accession number #GSE185317. For secondary analysis, we used our previously published NGS datasets, with accession numbers #GSE125405 and #GSE133500.

Field-specific reporting

Please select the one below that is the best fit for your research. If you are not sure, read the appropriate sections before making your selection.

[x] Life sciences [ ] Behavioural & social sciences [ ] Ecological, evolutionary & environmental sciences

For a reference copy of the document with all sections, see nature.com/documents/nr-reporting-summary-flat.pdf

Life sciences study design

All studies must disclose on these points even when the disclosure is negative.

Sample size	The overall experimental design has been apprpved and sample size has been deterimined by a prior analysis conducted by a biostatistician. In brief, sample size calculation was based on the expectated, biologically relevant difference in the measured parameter between the control and the test group (e.g., vehicle vs. treated; young vs. adult, etc.). Assuming normally distributed data from these quasi-continuous endpoints with a power of 80% and a two-sided type 1 error of 5%, the t-test for parallel groups was used to estimate the number of cases. Due to its multi-factorial setting, the experiments are evaluated with methods of analysis of variance (ANOVA) in the case of steady, normally distributed target variables. Corresponding non-parametric methods (e.g., Kruskal-Wallis) were used for data that are not normally distributed. The t-test or the Wilcoxon test for unrelated samples was used for two-group comparisons. Qualitative parameters are evaluated with the chi-square test or the exact test according to Fisher. Before the start of the statistical test evaluation, all parameters were descriptively analyzed according to their characteristics.
Data exclusions	Prior analysis exlclusion: experimental animals were excluded from the study based on a priori described featrues (e.g., apparent sickness, excessive weight loss, in-cage aggression, etc.). In case of human subjects, a priori exclusion criteria included chronic inflammatory disease, actue infection, surgical complication (e.g., excessive bleeding). Following data collection data were excluded only if a justified technical issue during data collection was proven.
Replication	Key exeprimments have been replicated by two independent researchers with similar outcome. Assays presented in the manuscript have been replicated successfully at least two independent times.
Randomization	Animals were randomized to assign them to control and test groups in the case of intervention studies (i.e., treating animals with vehicle or test compounds or high fat diet), by randomly selecting their numerical identification numbers. These identification numbers were generated using their cage identification numbers and date of birth. In case of human subjects we did not conduct intervention studies, and human fat samples were analysed on the day of surgery of the donor patients. Order of surgery was determined by the surgical team, and all patients at our surgery departments - apart from those who met some exlcuison criteria - were approached by the surgeon with the possibility of participating in the study, giving no reason for further randomizing samples.
Blinding	All samples were labeled with numerical codes, allowing blinding in data analysis, and investigators were blinded to gorup allocation during data collection.

Reporting for specific materials, systems and methods

We require information from authors about some types of materials, experimental systems and methods used in many studies. Here, indicate whether each material, system or method listed is relevant to your study. If you are not sure if a list item applies to your research, read the appropriate section before selecting a response.

Materials & experimental systems

n/a	Involved in the study	
[ ]	[x]	Antibodies
[ ]	[x]	Eukaryotic cell lines
[x]	[ ]	Palaeontology and archaeology
[ ]	[x]	Animals and other organisms
[ ]	[x]	Human research participants
[ ]	[x]	Clinical data
[x]	[ ]	Dual use research of concern

Methods

n/a	Involved in the study	
[x]	[ ]	ChIP-seq
[ ]	[x]	Flow cytometry
[x]	[ ]	MRI-based neuroimaging

Antibodies

Antibodies used	The complete antibody list is provided in Supplementary Table 2, along with their source, clone number and dilution.
Validation	Manufacturers of the used antibodies (listed in Supplementary Table 2) provided validation data of each antibody. We further performed postive and negative staining assays as shown in Extended Data Figure 8. In brief, as IRF7 positive control we used hematopoietic foci of newborn liver specimens, for UCP1 mouse interscapular adipose tissue specimens. As negative control we omitted primary antibodies in the staining procedure. We also used adipose tissue from IRF7 deficient adult mice for IRF7 immunostaining as negative control. Further validation of UCP1 labeling is provided in Cells

. 2021 Sep 9;10(9):2368. doi: 10.3390/cells10092368.

Eukaryotic cell lines

Policy information about cell lines

Cell line source(s)	3T3-L1 and THP-1 cell lines were obtained from ATCC.
Authentication	Expression of characteristic mRNA transcripts (e.g., Ifi204, Ifi203, Ifi202b of BALB/C origin, Fabp4 and Fasn for lineage identity; morphology (with phase contrast and with transmission electron microscopy) and cell-line specific behavior (i.e., adipogenic differentiation) have been tested for 3T3-L1 cells. For THP-1 cells morphology (monocytic or induced adheren macrophage-like), activation in response to stimuli were used to ensure lineage identity.
Mycoplasma contamination	The cell lines were regularly tested negative for Mycoplasma.
Commonly misidentified lines (See ICLAC register)	Not applicable (our cell lines are not listed in ICLAC register, as of October 26, 2021).

Animals and other organisms

Policy information about studies involving animals; ARRIVE guidelines recommended for reporting animal research

Laboratory animals	mouse, Mus musculus, C57BL/6 as wild-type, MDA5-KO and IRF7-KO on a C57BL/6 background, RIG-I-KO on CD1 background (for this we used CD1 as control). Housing conditions: 12h/12h day/might cycle, under SPF health monitoring, in individually ventilated cages, at ambient temperature (20-22°C) and at 55% air humidity. Animals were housed 1 animal per cage (during high-fat diet and indirect calorimetry) or in groups with bedding and paper nesting material. The animals could move freely. The cages stood next to each other so that the animals have visual contact. The animals were given ad libitum access to water and food. As standard protocol at Ulm University, the requirements of ETS123 were met.
Wild animals	none
Field-collected samples	none
Ethics oversight	Research involving animals was approved by the regional governmental ethics and animal welfare committee in Tübingen, Germany (#1511; #1557; #1492; #1546; #o.232-1,2,4,5). Study approvals are available upon request in German.

Note that full information on the approval of the study protocol must also be provided in the manuscript.

Human research participants

Policy information about studies involving human research participants

Population characteristics	Study Population: children and adolescents undergoing elective surgery at the University Hospital Leipzig, Germany and Pediatric Surgery Department, Institute of Pediatrics, University of Debrecen, Hungary, between 2010 and 2022.<br>Indication of surgery: retentio testis, hydrocele, hernia, collar fistula, naevus removal, stoma closure, gynecomastia,anal fistula, other<br><br>The study cohort may be stratified for lean (definded as BMI <1.28 SDS) and overweight/obese (definded as BMI>= 1.28 SDS) children for posthoc analyses, no intervention.<br><br>Inclusion Criteria:<br>1. body weight: 3000 g - 180 kg<br>2. suitable surgical procedure<br>3. signed informed consent by the guardians and the study participant, if older 12 years (Germany), or signed informed consent by the guirdans of the study participants under 18 years of age (Hungary)<br><br>Exclusion Criteria:<br>1. severe chronic and inflammatory diseases<br>2. acute or chronic infections<br>3. oncological diseases<br>4. clotting disorders<br>5. complications during surgical procedure<br>6. drug treatment<br><br>Age range of participants: 0-18 years<br>Sex of participants: male (65%), female (35%). The higher percentage of male participants was due to the higher rate of medical conditions among males in pediatric surgery practice (i.e., surgical resolution of retentio testis or testicular torsion apply only to boys, and inguinal hernia is more common among boys than girls).
Recruitment	Participants underwent elective surgery at University Hospital of University of Leipzig, Germany between 2010 and 2020 or at Pediatric Surgery Department, Institute of Pediatrics, University of Debrecen, Hungary in 2022 (children and adolescents). Recruitment from 2010 to 2022. Data collection for Illumina and histology/FACS/immunofluorescence analysis: 2010-2021,

functional assays/FACS: 2022. Parents or guardians of the patients were contacted by the surgeon before the surgery, providing informed consent abut the study. There is self-selection bias involved, as participants did not actively apply for participation in the study. Since each patient in the indicated time period of recruitment was approcahed with the information on our study (unless an exclusion criteria was apparent for the surgeon), there is no recruitment bias. Parents/guardians were free to accept or decline the participation in the study, and they receibed sufficient time to make a decision. The study cohort may be stratified for lean (definded as BMI <1.28 SDS) and overweight/obese (definded as BMI>= 1.28 SDS) for posthoc analyses, no intervention. Patient selection was not affected by the BMI SDS, gender or other clinical parameter, to avoid recruitment bias.

Ethics oversight

The study protocol was approved by the local ethics committee of the Medical Faculty, University of Leipzig (#265-08-ff; NCT02208141) and the Medical Faculty of University of Debrece (#RKEB6057).

Note that full information on the approval of the study protocol must also be provided in the manuscript.

Clinical data

Policy information about clinical studies

All manuscripts should comply with the ICMJEguidelines for publication of clinical research and a completedCONSORT checklist must be included with all submissions.

Clinical trial registration

NCT02208141

Study protocol

https://clinicaltrials.gov/ct2/show/NCT02208141

Data collection

Participants of this study were undergoing elective surgery at the University Hospital Leipzig, Germany and Pediatric Surgery Department, Institute of Pediatrics, University of Debrecen, Hungary, between 2010 and 2022. Recruitment period was between 2010-2022. Parents/guirdans of each eligible patient was approached by one member of the surgical team, to prvide information about our study, answer questions of the parents/guirdans, and ensure that all necessary documents (informed consent, data protection, etc.) are signed and archived. There is no self-selection bias in the study participation.

Outcomes

Primary Outcome Measures :

Adipose tissue dysfunction [Time Frame: 10 years]

Adipose tissue dysfunction is assessed by evaluation of adipocyte size and number (hypertrophy vs. hyperplasia), adipocyte proliferation and differentiation, (lipid) cellular metabolism, inflammation, gene expression, fibrosis, and others; the association of AT dysfunction with clincial phenotype will be assessed

Secondary Outcome Measures :

Presence of beige/brown adipose tissue (BeAT/BAT) [Time Frame: 10 years]

Adipose tissue samples are evaluated for the presence of BeAT/BAT on histological and molecular level and association with clinical phenotype isinvestigated

Inflammation of adipose tissue [Time Frame: 10 years]

Inflammation is assessed by evaluating macrophage infiltration on histological and molecular expression level. Also, association with clinical phenotype is assessed.

Biospecimen Retention (Leipzig only):

samples with DNA

adipose tissue sample

mRNA from adipose tissue samples, adipocytes, stromal vascular fraction

protein from whole adipose tissue samples

blood samples for DNA analysis, serum samples

urine samples

Biospecimen Retention (Debrecen only):

adipose tissue sample

patient charts (follow-up of clinical phenotype)

Flow Cytometry

Plots

Confirm that:

- [x] The axis labels state the marker and fluorochrome used (e.g. CD4-FITC).
- [x] The axis scales are clearly visible. Include numbers along axes only for bottom left plot of group (a 'group' is an analysis of identical markers).
- [x] All plots are contour plots with outliers or pseudocolor plots.
- [x] A numerical value for number of cells or percentage (with statistics) is provided.

Methodology

Sample preparation	Fat samples were digested with collagenase, followed by separation of cell fractions and subsequently analyzed or cultured, as described: Ampem G, and Röszer T. In: Badr MZ ed. Nuclear Receptors: Methods and Experimental Protocols. New York, NY: Springer New York; 2019:225-36. ATMs were purified before FACS analysis with magnetic bead labeling and sorting, as described in the Supplementary Information.
Instrument	BD LSR II
Software	FlowJo v10, FACSDiva 8.01
Cell population abundance	2,000-10,000 cells.
Gating strategy	Single cell events are gated first, adipocyte and ATM populations second (based on SSC-A and FSC-A parameters), this is fllowed by gating for primary antibody signals (e.g., F4/80). Details provided in reference: Ampem G, and Röszer T. In: Badr MZ ed. Nuclear Receptors: Methods and Experimental Protocols. New York, NY: Springer New York; 2019:225-36., also shown in SFig. 15D

☒ Tick this box to confirm that a figure exemplifying the gating strategy is provided in the Supplementary Information.
